# Regulation of the p75 neurotrophin receptor attenuates neuroinflammation and stimulates hippocampal neurogenesis in experimental *Streptococcus pneumoniae* meningitis

**DOI:** 10.1186/s12974-021-02294-w

**Published:** 2021-11-02

**Authors:** Dandan Zhang, Shengnan Zhao, Zhijie Zhang, Danfeng Xu, Di Lian, Jing Wu, Dake He, Kun Sun, Ling Li

**Affiliations:** 1 Department of Pediatric Neurology, Xinhua Hospital Affiliated to Shanghai Jiao Tong University School of Medicine, Kongjiang Road 1665, Shanghai, 200092 China; 2 Department of Pediatric Cardiology, Xinhua Hospital Affiliated to Shanghai Jiao Tong University School of Medicine, Kongjiang Road 1665, Shanghai, 200092 China

**Keywords:** *Streptococcus pneumoniae* meningitis, Brain injury, p75 neurotrophin receptor, Neuroinflammation, Hippocampal neurogenesis

## Abstract

**Background:**

*Streptococcus pneumoniae* meningitis is a destructive central nervous system (CNS) infection with acute and long-term neurological disorders. Previous studies suggest that p75NTR signaling influences cell survival, apoptosis, and proliferation in brain-injured conditions. However, the role of p75NTR signaling in regulating pneumococcal meningitis (PM)-induced neuroinflammation and altered neurogenesis remains largely to be elucidated.

**Methods:**

p75NTR signaling activation in the pathological process of PM was assessed. During acute PM, a small-molecule p75NTR modulator LM11A-31 or vehicle was intranasally administered for 3 days prior to *S. pneumoniae* exposure. At 24 h post-infection, clinical severity, histopathology, astrocytes/microglia activation, neuronal apoptosis and necrosis, inflammation*-*related transcription factors and proinflammatory cytokines/mediators were evaluated. Additionally, p75NTR was knocked down by the adenovirus-mediated short-hairpin RNA (shRNA) to ascertain the role of p75NTR in PM. During long-term PM, the intranasal administration of LM11A-31 or vehicle was continued for 7 days after successfully establishing the PM model. Dynamic changes in inflammation and hippocampal neurogenesis were assessed.

**Results:**

Our results revealed that both 24 h (acute) and 7, 14, 28 day (long-term) groups of infected rats showed increased p75NTR expression in the brain. During acute PM, modulation of p75NTR through pretreatment of PM model with LM11A-31 significantly alleviated *S. pneumoniae-*induced clinical severity, histopathological injury and the activation of astrocytes and microglia. LM11A-31 pretreatment also significantly ameliorated neuronal apoptosis and necrosis. Moreover, we found that blocking p75NTR with LM11A-31 decreased the expression of inflammation*-*related transcription factors (NF-κBp65, C/EBPβ) and proinflammatory cytokines/mediators (IL-1β, TNF-α, IL-6 and iNOS). Furthermore, p75NTR knockdown induced significant changes in histopathology and inflammation*-*related transcription factors expression. Importantly, long-term LM11A-31 treatment accelerated the resolution of PM-induced inflammation and significantly improved hippocampal neurogenesis.

**Conclusion:**

Our findings suggest that the p75NTR signaling plays an essential role in the pathogenesis of PM. Targeting p75NTR has beneficial effects on PM rats by alleviating neuroinflammation and promoting hippocampal neurogenesis. Thus, the p75NTR signaling may be a potential therapeutic target to improve the outcome of PM.

**Supplementary Information:**

The online version contains supplementary material available at 10.1186/s12974-021-02294-w.

## Background

Bacterial meningitis (BM) is a serious infection of the central nervous systems (CNS) with high morbidity and mortality worldwide. *Streptococcus pneumoniae* and *Neisseria meningitidis* are the leading pathogens of childhood meningitis beyond neonatal age [1, 2]. Pneumococcal meningitis (PM) causes long-term neurological defects than other BM pathogens, about 50% of the survivors have persistent neurological sequelae throughout their lives, including hearing impairment, learning, memory disorders and seizures [3]. During PM, *S. pneumoniae* invades the subarachnoid space with various virulence factors, and the brain-resident immune cells together with the recruited neutrophils produce a severe inflammatory response. The direct neurotoxicity produced by *S. pneumoniae* and the innate and adaptive inflammatory response caused by immunocompetent cells after recognition of bacteria together can severely PM-related central and peripheral nervous systems [4, 5]. PM-induced CNS injury is characterized by cortical necrosis and neuronal apoptosis in the hippocampal dentate gyrus (DG), which is closely related to learning and behavioral deficits [6–9]. The main challenges for fighting against *S. pneumoniae* include the rising of antibiotic resistance and the serotype replacement phenomenon of vaccination [10–12]. Although corticosteroids have been recommended as an effective anti-inflammatory adjuvant treatment for PM, no beneficial effect has been observed in pediatric PM [13]. Aggravate hippocampal apoptosis with adjuvant dexamethasone was reported in some experimental studies [14, 15]. Hence, we should strive to find alternative adjuvant therapies to reduce the burden of pediatric PM.

Brain-derived neurotrophic factor (BDNF) is a key member of the neurotrophic family that is widely expressed in the CNS, which plays a critical role in the survival, development, and differentiation of neurons through activation of its high-affinity binding to tropomyosin-receptor kinase B (TrkB) [16, 17]. The neuroprotective effect of BDNF on BM has been confirmed in various clinical and experimental studies. Our group has been committed to study the role of BDNF/TrkB interaction in the course of PM. In previous studies, we reported that the levels of BDNF and its receptor TrkB were upregulated following acute PM, exogenous BDNF adjuvant therapy could reduce neuroinflammation, protect the survival of neurons in the cortex and hippocampus, and improve auditory function [18–21]. Additionally, we also found that the long-term administration of exogenous BDNF promoted the neurogenesis of neural stem cells (NSCs) in the hippocampus after PM; however, few NSCs differentiated into mature neurons [22]. The underlying mechanism is still unclear. It is noteworthy that the mature forms of BDNF activate survival and growth signals by activating TrkB; however, its precursor (proBDNF), binds to tumor necrosis factor receptor-like molecule p75 neurotrophin receptor (p75NTR), display an opposing biological activity to mature BDNF by inducing neuronal apoptosis and death [23–25].

p75NTR is a member of the tumor necrosis factor (TNF) receptor superfamily, which plays a key role in the proneurotrophin mediated multiple pathways, including apoptosis, death and cell degeneration [26]. An increasing number of studies reported that the interaction of p75NTR with proneurotrophins is upregulated after various forms of CNS injury. p75NTR-null mice displayed reduced brain injuries and motor deficits after traumatic brain injury (TBI), attenuated the hyperphosphorylation of Tau in the pR5 model, improved spatial learning and enhanced long-term potentiation (LTP) [27–29]. Consistently, overexpression of p75NTR negatively modulated dendritic complexity and spine density in hippocampal neurons [30], and reproduced learning and memory deficits in WT mice with Huntington’s disease (HD) [31]. Moreover, it is reported that p75NTR regulated p35/CDK5 signaling, promoting neuronal apoptosis after intracerebral hemorrhage (IHC) [32]. Of note, evidences have shown that the expression of p75NTR is associated with neuroinflammation [33, 34]. p75NTR blockade reduces microglial activation in subarachnoid hemorrhage and Parkinson’s disease [35, 36]. In such scenario, pharmacological modulators targeting p75NTR have gradually become an important neuroprotective strategy after brain injury. LM11A-31, non-peptide, a small-molecule p75NTR modulator, show excellent blood–brain barrier penetration and tolerability and mitigates pathology in models of Alzheimer’s disease, Huntington’s disease and TBI injury. In the Alzheimer model mice model, LM11A-31 significantly reduced neuroinflammation responses and cholinergic degeneration, with subsequent improved cognitive deficits [37]. Normalizing p75NTR with LM11A-31 restored pro-survival signaling while inhibiting degenerative signaling and decreased inflammation and restored synaptic plasticity and memory in HD mouse models [38]. In addition to its neuroprotective effects, LM11A-31 has been reported to potently improve hippocampal neurogenesis by promoting progenitor cell survival and proliferation in TBI injury [39]. While these findings provide a solid rationale for therapeutic targeting of p75NTR in inflammatory injury and degenerative disease of CNS; however, the role of P75NTR in the pathological process of PM has not been elucidated. To the best of our knowledge, this is the first study showing that modulation of the p75NTR improves *S. pneumoniae-*induced brain injury, including anti-inflammation and promotes hippocampal neurogenesis.

In the present study, we investigated whether proBDNF/p75NTR interaction participated in the pathological process of PM. Moreover, pharmacological regulation using LM11A-31 or genetic silencing of p75NTR could reduce neuroinflammation in the PM rat model. We further examined whether long-term administration of LM11A-31 after PM could improve hippocampal neurogenesis.

## Methods

### Infecting organisms

The standard strain of serotype III *S. pneumoniae* from American Type Culture Collection (ATCC, Manassas, VA, USA) was cultured on blood agar plates for 18 h and then transferred into Vital Aer Broth (R&D Systems, Minneapolis, MN, USA) for another 18 h at 37 °C in air with 5% CO_2_ to achieve the logarithmic growth phase. Then, the bacteria were centrifuged for 20 min at 5000×*g*, washed twice, and resuspended in sterile saline to approximate 1 × 10^4^ colony forming units (CFU)/ml using a nephelometer (bio-Merieux, Marcy-l’Étoile, France).

### Rat model of *S. pneumonia* meningitis

Three-week-old Sprague–Dawley rats were obtained from the Shanghai Laboratory Animal Management Center (Shanghai, China). A well-established rat model of pneumococcal meningitis was used as described previously [22, 40]. In brief, rats were anesthetized with pentobarbital sodium (50 mg/kg), 30 μL cerebrospinal fluid (CSF) was slowly removed via intracisternal puncture. Then, infected intracisternal with 30 μL volume containing either 1 × 10^4^ CFU/mL *S. pneumoniae* or pyrogen-free saline. At 24 h post-infection, rats were weighed and a clinical score evaluated the severity of the disease (1 = coma, 2 = does not turn upright, 3 = turns upright within 30 s; 4 = turns upright within < 5 s, and 5 = normal) in a blinded manner. Animals were sacrificed and perfused with pyrogen-free saline. The brain tissues were removed and separated immediately, half of the hemispheres were frozen in liquid nitrogen and half of the hemispheres were fixed in 4% paraformaldehyde. Samples of cerebellar and spleen homogenates were plated in serial dilutions on the sheep blood agar plates under 37 °C and 5% CO_2_ overnight to determine the bacterial titers.

### Experimental design and drug injection

**Fig. 1:**
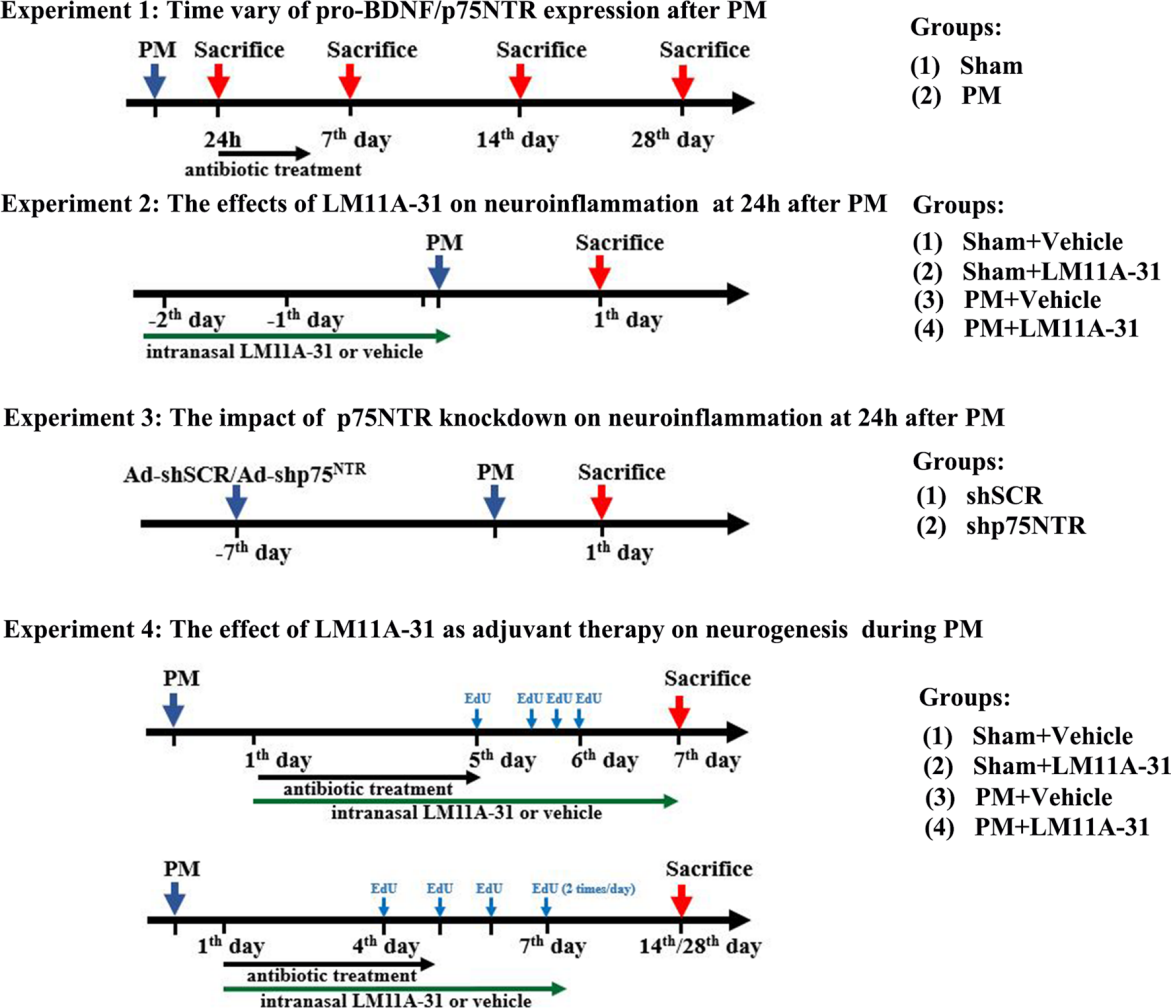
Timeline of different treatments and animal groups

A total of 172 rats were included in this study, which were randomly divided into the following experimental, as shown in Fig. 1.

In the first experiment, 36 animals were assigned to two groups: Sham group and PM group. All rats from day 7, 14 and 28 groups received antibiotic treatment (100 mg/kg ceftriaxone; Beyotime Biotech, Shanghai, China) continuously through subcutaneous injection for 5 days, starting from 24 h post-infection. Rats were sacrificed after 24 h (*n* = 6 each), days 7 (*n* = 4 each), 14 (*n* = 4 each), or 28 (*n* = 4 each) to evaluate the expression of proBDNF/p75NTR.

In the second experiment, 60 animals were divided into four groups: Sham+Vehicle (water, 60 μL/day) group (*n* = 10), Sham+LM11A-31 (15 μg/60 μL/day) group (*n* = 10), PM+Vehicle group (*n* = 20) and PM+LM11A-31group (*n* = 20). LM11A-31(2-amino-3-methyl-*N*-[2-(4-morpholinyl)ethyl]-pentanamide) was obtained from MedChemExpress (Monmouth Junction, NJ, USA). For intranasal compound delivery, LM11A-31 was dissolved in water at a concentration of 0.25 mg/ml, instilled in alternating nares for 2 min, repeated five times per nares, for 60 μl (15 μg of the compound). Animals were pretreated once daily for three consecutive days of LM11A-31 in water or water alone, and the PM model mentioned above was constructed 30 min after the last pretreatment. At 24 h post-infection, rats were scarified to investigate brain injury and neuroinflammation during acute PM.

In the third experiment, 20 animals were divided into two groups: shSCR group (*n* = 10) and shp75NTR group (*n* = 10). p75NTR knockdown was performed by RNA interference using adenovirus transfection. The shRNA sequence targeting p75NTR (shp75NTR, 2 × 10^10^ pfu/mL) and a scrambled sequence (shSCR, 2.4 × 10^10^ pfu/mL) used as a nontargeting shRNA control were supplied by Genechem Co. Ltd. (Shanghai, China). The sequences used were as follows: 5′- GAGGTGCCAAGGAGACATGTT -3′ for p75NTR, and 5′- TTCTCCGAACGTGTCACGT -3′ for scrambled sequence. Control adenovirus was diluted to 2 × 10^10^ pfu/mL in enhanced transfection solution (GeneChem) before intracerebroventricular injection. Rats were stereotactically injected with 3 μL adenovirus solution (2 × 10^10^ pfu/mL) with shp75NTR or shSCR into the right lateral ventricle at a rate of 0.2 μL/min with 10 μL-Hamilton syringe 7 days before PM. The stereotaxic coordinates were 3.8 mm rostral to the lambdoid suture of the skull, 2 mm lateral to the right side from the midline of the skull, and 2.5 mm from the skull surface. At 24 h post-infection, rats were studied to assess the effect of p75NTR knockdown on neuroinflammation.

In the final experiment, 56 animals were divided into four groups: Sham+Vehicle (water, 60 μL/day) group, Sham+LM11A-31 (15 μg/60 μL/day) group, PM+Vehicle group and PM+LM11A-31 group. Rats from all groups received antibiotic treatment in line with the first experiment. Meanwhile, rats were treated once daily for consecutive 7 days with LM11A-31 in water, or water alone, starting 24 h after infection. The dose and mode of administration were the same as in the second experiment. On day 7 (*n* = 18), 14 (*n* = 18), or 28 (*n* = 20), animals were sacrificed to evaluate neuroinflammatory changes and hippocampal neurogenesis.

### Ethynyl deoxyuridine treatment

Ethynyl deoxyuridine (EdU) acts as a marker of dividing cells to monitor neurogenesis. To evaluate cell proliferation, EdU (50 mg/kg; Santa Cruz Biotechnology, Dallas, TX, USA) diluted in 5% DMSO was given every 4 h, starting from 30 h before sacrifice on day 7 post-infection, a total of four times. Animals were sacrificed 18 h after the last EdU treatment. To evaluate the differentiation of NSCs, animals were injected with the same dose of EdU twice daily on days 4–7 after infection, and animals were sacrificed on day 14 or 28 post-infection.

### Western blot analysis

The brain tissues (cortex and hippocampus) lysates were homogenized in cold radioimmunoprecipitation assay (RIPA) buffer containing a protease inhibitor cocktail (#87786, Thermo Scientific, Waltham, MA, USA). Total protein was quantified using the BCA assay reagent (#23227, Thermo Fisher Scientific, Waltham, MA, USA). Aliquots (60 μg total proteins) were loaded into SDS–polyacrylamide gels and transferred to polyvinylidene fluoride (PVDF) membranes. After blocking in 5% fat-free milk (#9999S, Cell Signaling Technology, Danvers, MA, USA), the membranes were incubated overnight at 4 °C with the following primary antibodies: mouse anti-β-actin (1:1000, #3700, Cell Signaling Technology, Danvers, MA, USA), rabbit anti-proBDNF (1:1000, #PA5-77533, Thermo Fisher Scientific, Waltham, MA, USA), rabbit anti-p75NTR (1:1000, #4201, Cell Signaling Technology, Danvers, MA, USA), rabbit anti-NF-kB p65 or phospho-NF-kB p65 (1:1000, #8242, #3033, Cell Signaling Technology, Danvers, MA, USA) and rabbit anti-C/EBPβ (1:1000, #ab32358, Abcam, Cambridge, UK). Primary antibodies were detected with horseradish peroxidase-conjugated IgG secondary antibody, then exposed to a chemiluminescence system and visualized using the ChemiDOC XRS+ imaging system (BIO-RAD, Hercules, CA). Protein expression was normalized to the same sample expression of β-actin. The relative band intensity was quantified using ImageJ software (National Institutes of Health, USA).

### Tissue pathology, Fluoro-Jade B and terminal deoxynucleotidyl transferase dUTP-nick-end labeling staining

Animals were anesthetized and perfused through the heart with sterile saline 24 h after infection. Then, brains were fixed in 4% paraformaldehyde at 4 °C. After 24 h of fixation, the brain tissues were embedded in paraffin wax on the oriented edge and cut into coronal 5-μm-thick sections for hematoxylin and eosin (H&E) staining. The TdT-mediated dUTP nick end labeling (TUNEL) assay was performed using the In Situ Cell Death Detection kit (Roche, Basel, Switzerland), according to the manufacturer’s instructions. In brief, deparaffinized tissue sections were incubated with proteinase K (20 µg/mL) for 15 min at room temperature and immersed in 3% H_2_O_2_ in methanol for 10 min. Permeabilization with 0.3% Triton-X-100 for 10 min was performed on ice. The sections were incubated with the TUNEL reaction mixture at 37 °C in a humidified chamber for 2 h, then incubated with fresh prepared 4′,6-diamidino-2-phenylindole (DAPI, Vector Laboratories, Burlingame, CA) reagent for 10 min in a dark room. For Fluoro-Jade B (FJB) staining, deparaffinized tissue sections were stained with 0.25% (50% glacial acetic acid as solvent) FJB overnight at 4 °C and nuclear staining with DAPI the next day following product instructions.

### Immunofluorescence

5-μm-thick paraffin-embedded sections of the brain were prepared as described above. To determine p75NTR expression patterns in different kinds of cells in the brain, double-labeling immunofluorescence with p75NTR&NeuN, p75NTR &Iba-1, p75NTR &GFAP was performed in rats after 24 h infection. Immunofluorescence single-labeling immunofluorescence was used to detect the activation of astrocytes and microglia in the cerebral cortex and hippocampus. In addition, for immunofluorescence evaluation of EdU^+^ and DCX^+^, NeuN^+^ cells in the hippocampal DG, on days 7 and 14 post-treated with LM11A-31, EdU&DCX was used to determine neuronal progenitor cells proliferation. On day 28 post-treated with LM11A-31, EdU&NeuN was used to determine neurogenesis. The brain sections were permeabilized for 10 min with 0.3% Triton-X100, and blocked with 5%BSA for 1 h at room temperature. The following primary antibodies: rabbit anti-p75NTR (1:200, #ab52987, Abcam, Cambridge, UK), mouse anti-NGFR p75 (1:200, sc-271708, Santa Cruz Biotechnology, Dallas, TX, USA), goat anti-Iba-1 (1:500, #ab5076 Abcam, Cambridge, UK), mouse anti-GFAP (1:500, #GB12096, Servicebio, Wuhan, China), rabbit anti-DCX (1:500, #GB11317, Servicebio, Wuhan, China) and rabbit anti-NeuN (1:500, #ab177487, Abcam, Cambridge, UK) incubated slices overnight at 4 °C. After washing three times with PBS, slices were incubated with secondary antibodies for 1 h at room temperature in the dark, and then mounted with DAPI reagent to stain for nuclei. To detect EdU staining, sections were incubated with an EdU imaging kit (#C10310-1, Ribobio, Guangzhou, China) followed after washing the secondary antibody with PBS. Then, stained sections were rinsed thoroughly with PBS and observed by a fluorescence microscope (Nikon, Japan).

### Enzyme-linked immunosorbent assay (ELISA)

Blood samples were collected and centrifuged at 4 °C, 4500 rpm for 15 min to obtain the serum. The concentrations of interleukin (IL)-1β, tumor necrosis factor-α (TNF-α), IL-6, and inducible nitric oxide synthase (iNOS) in the serum were determined using ELISA kits (ELK Biotechnology, Wuhan, China) according to the manufacturer’s instructions. The results were expressed as pg/mL.

### RNA isolation and quantitative real-time PCR

Total RNA was isolated from brain tissues, including the cerebral cortex and hippocampus, with a Total RNA Kit (TaKaRa, Shiga, Japan, catalog #9767), following the manufacturer’s instructions. The RNA concentration was quantified using a NanoDrop 2000 (Thermo Fisher Scientific) and was reverse transcribed to cDNA using the PrimeScript™ RT Master Mix (TaKaRa, Shiga, Japan, catalog #RR036A). Quantitative real-time PCR was carried out with SYBR Premix Dimmer Eraser kit (TaKaRa, Shiga, Japan, catalog #RR420A) in 20 µl of final volume using QuantStudio 3 system (Applied Biosystems, Carlsbad, CA, USA). Primers for real-time PCR (RT-PCR) were designed using Premier 5 software, and the sequences are listed in Additional file 1: Table 1. The fold change of target genes was determined from the observed *Ct* (cycle threshold) values and calculated using the 2^−ΔΔCt^ method, and *β*-actin served as the reference gene.

### Cell Counting

In tissue sections, FJB‐positive cells, Iba-1-positive cells and GFAP-positive cells in cortex and hippocampus were observed from three randomly selected microscopic fields at ×200 magnification. Positive cells count was undertaken on a microscope (Nikon Eclipse C1, Japan) with digitalization software CaseViewer 2.0 (3D HISTECH, Hungary). Randomly selected three fields of view from coronal cortex and DG section to count the cell number, then averaged the results per group. For TUNEL-positive cells, EdU&DCX- and EdU&NeuN-labeled cells count in the hippocampus, coronal section of the DG (including adjacent sub-granular zone [SGZ], granule cell layer [GCL], and molecular layer [ML]) within hippocampus was analyzed from each section. We obtained the cell number of the DG from each animal. All counts were performed by the same observer who was blinded to experimental groups.

### Statistical analysis

All the data are presented as mean ± SEM. Differences between the two groups were detected by unpaired Student *t*-test (parametric data). Two-way ANOVA followed by Tukey’s post-hoc test was used to compare differences between multiple groups. The survival curve was performed using the Kaplan–Meier method and analyzed by the log-rank test. All statistical analyses were performed using GraphPad Prism 8 (GraphPad Software, San Diego, CA, USA). *p*-value < 0.05 was considered statistically significant.

## Results

### Increased p75NTR expression 24 h after PM and is mainly expressed in astrocytes and neuron cells

**Fig. 2:**
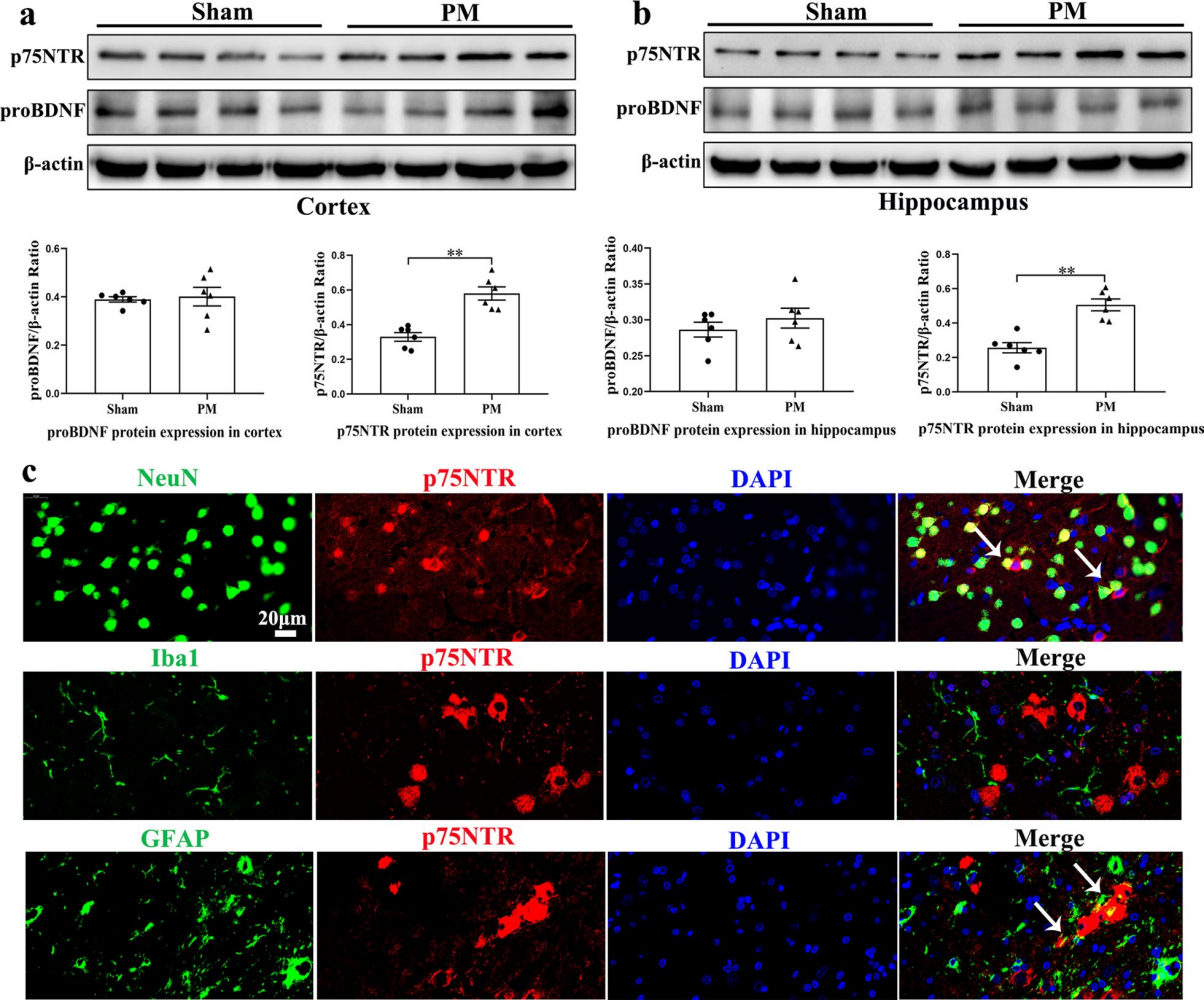
The expression of proBDNF/p75NTR and p75NTR cellular distribution at 24 h after PM induction. **a**, **b** Western blot analysis of proBDNF/p75NTR protein levels in the cortex and hippocampus at 24 h post-infection. Proteins were normalized to *β*-actin. **c** Representative immunofluorescence colocalization images of p75NTR/NeuN, p75NTR/Iba1, p75NTR/GFAP in the cortex at 24 h post-infection. Neuron cells, microglial cells, and astrocytes are labeled with NeuN (green), Iba-1 (green), and GFAP (green), respectively. (p75NTR = red and DAPI = blue). Data are presented as the mean ± SEM (*n* = 2–6 rats). **p < 0.01. Scale bar = 20 μm

To investigate the role of proBDNF/p75NTR in rats after PM, proBDNF and p75NTR protein expression in the cortex and hippocampus were examined by Western blot analysis. Following PM, p75NTR protein expression was significantly increased in both the cortex (*p* < 0.01; Fig. 2a) and hippocampus (*p* < 0.01; Fig. 2b) in PM group 24 h after infection. However, the expression of proBDNF protein in the cortex and hippocampus showed no obvious difference between the sham group and the PM group. To determine the cellular localization of p75NTR in infected brain tissues, we detected paraffin sections by double-labeling immunofluorescence. p75NTR was present in the NeuN-labeled neuron cells and GFAP-labeled astrocytes, but absent from microglial cells in the cortex (Fig. 2c).

### LM11A-31 pretreatment mitigates the clinical and pathological severity of rats at 24 h after PM

**Fig. 3:**
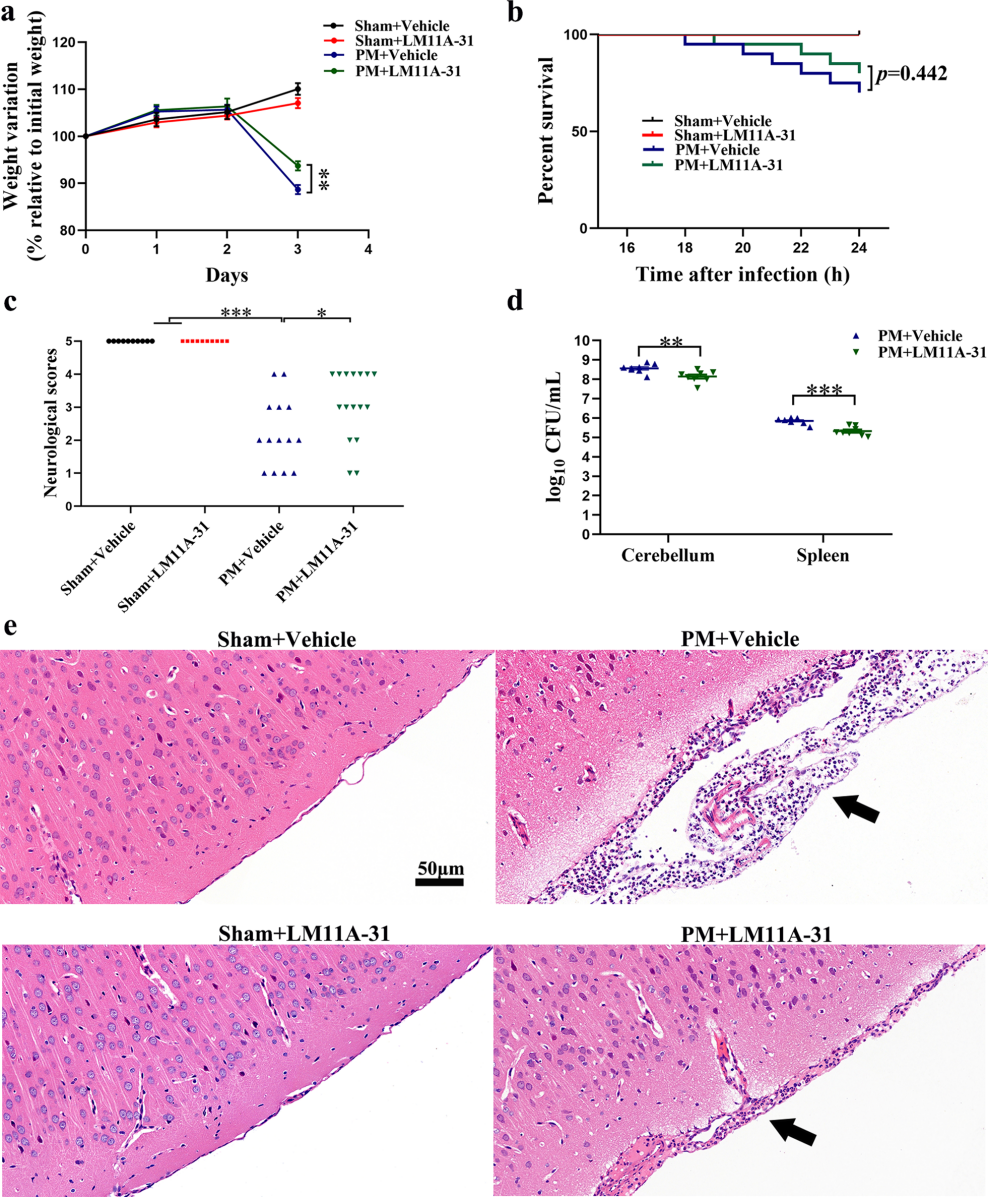
LM11A-31 pretreatment improves the clinical and pathological severity at 24 h after PM induction. **a** Weight loss, **b** survival, **c** neurological scores and **d** bacterial titer in different groups were evaluated at 24 h post-infection. **e** Representative images showing the histological H&E staining of the coronal brain sections. Data are presented as the mean ± SEM (*n* = 8–20 rats). *p < 0.05, **p < 0.01, ***p < 0.001. Scale bar = 50 μm

To determine whether the p75NTR signaling pathway participates in the PM pathogenesis, regulates p75NTR can directly affect the clinical and pathological severity post-infection, the rats were pretreated with a small-molecule p75NTR signaling modulator, LM11A-31, once daily for 3 consecutive days prior to *S. pneumoniae* inoculation. All rats were weighed from the 1st day of LM11A-31 or vehicle pretreatment, the changes in body weight of rats were shown in Fig. 3a. Before infection, the bodyweight of rats in all groups increased steadily, the differences between vehicle pretreatment and LM11A-31 pretreatment were not statistically significant. All rats from PM+Vehicle group loss weight 24 h after infection than the sham group (*p* < 0.001 vs Sham+Vehicle,* p* < 0.001 vs Sham+LM11A-31). In both infection groups, the rats pretreated with LM11A-31 showed a slighter decrease of bodyweight than the rats in the PM+Vehicle group (*p* < 0.01). The survival of rate was markedly reduced in infected rats compared to sham rats. However, LM11A-31 pretreatment did not significantly improve survival in infected animals (*p* = 0.442, Fig. 3b). As shown in Fig. 3c, upon infection, clinical scores were considerably decreased in infected animals compared to rats in the sham group. Clinical scores of infected rats pretreated with LM11A-31 were significantly higher than those in the PM+Vehicle group (*p* < 0.05) at 24 h after infection. At 24 h post-infection, all infected rats exhibited positive bacterial cultures from cerebellar and spleen homogenate (Fig. 3d). Moreover, bacterial concentrations were significantly reduced in rats pretreated with LM11A-31 in the cerebellar (*p* < 0.01) and spleen (*p* < 0.001). The histopathological changes within the brain tissue in all groups of rats are shown in Fig. 3e. Subarachnoid expansion and a high number of inflammatory cells infiltration were observed in all infected groups. Additionally, animals pretreated with LM11A-31 exhibited lesser inflammatory cells infiltration than observed in the PM+Vehicle group. These results indicate that intranasal administration of LM11A-31 can effectively regulate the role of p75NTR in mediating PM-induced brain injury.

### LM11A-31 pretreatment suppresses the activation of microglia and astrocytes in the cortex and hippocampus of rats at 24 h after PM

**Fig. 4:**
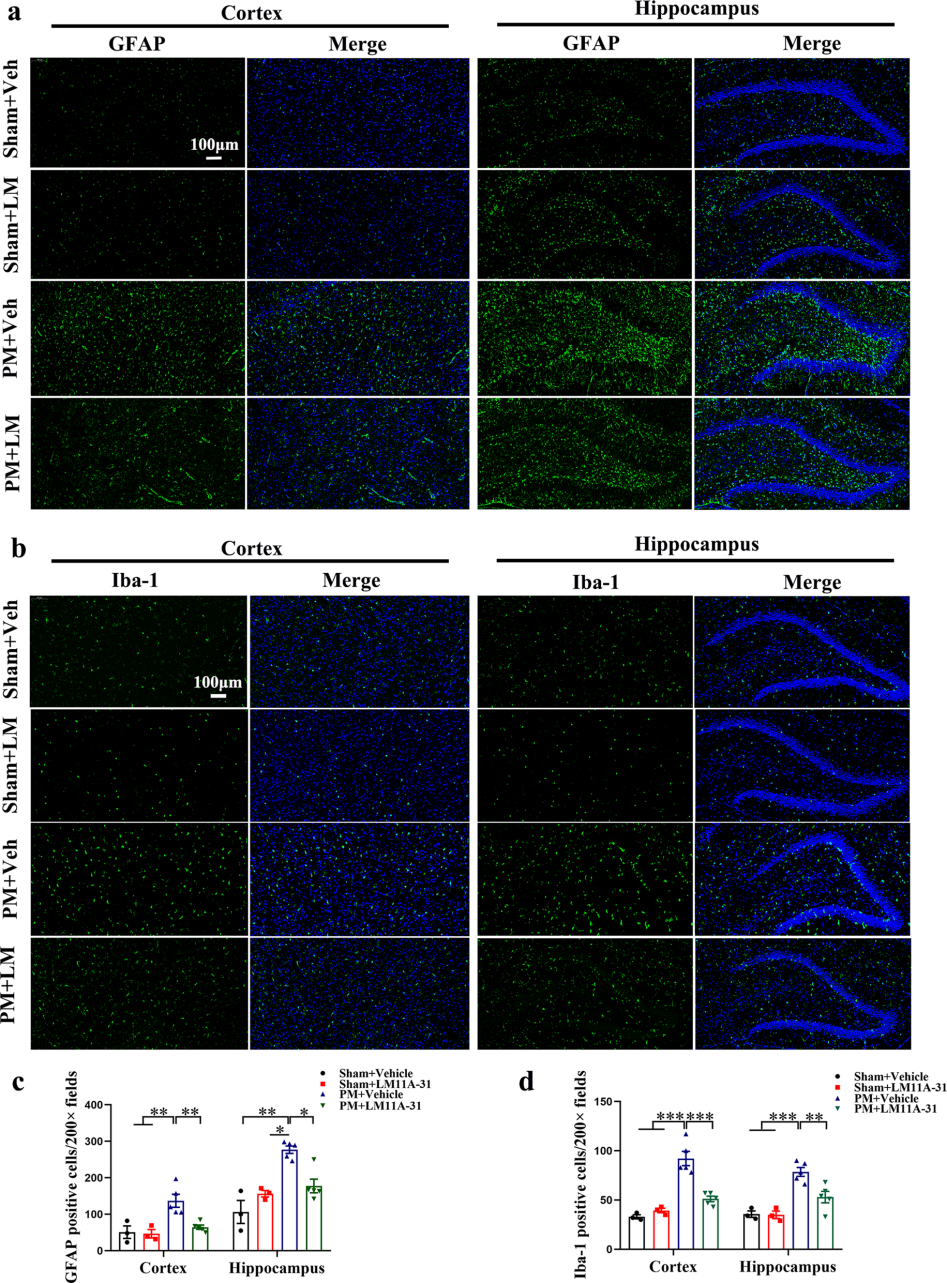
LM11A-31 pretreatment suppresses the activation of microglia and astrocytes at 24 h after PM induction. Immunofluorescence analysis for GFAP-positive cells and Iba-1-positive cells in cortex and hippocampus of rats in different groups. **a**, **b** Representative images stained with GFAP (green) and Iba-1 (green) in the cortex and hippocampus at 24 h post-infection. **c**, **d** The number of GFAP-positive cells/200× fields and Iba-1-positive cells/200× fields in the cortex and hippocampus at 24 h post-infection. Data are presented as the mean ± SEM (*n* = 3–5 rats). *p < 0.05, **p < 0.01, ***p < 0.001. Scale bar = 100 μm

The occurrence and development of PM are closely related to the overamplification of neuroinflammatory networks mainly mediated by astrocytes and microglia. We assessed the levels of astrocyte (GFAP) and microglial (IBA-1) markers 24 h post-infection to observe the activation of glial cells in the brains of rats in each group. As shown in Fig. 4a, we found significantly increased expression of astrocyte markers GFAP in the cortex in the PM+Vehicle group (*p* < 0.01 vs Sham+Vehicle, *p* < 0.01 vs Sham+LM11A-31, Fig. 4c) and hippocampus (*p* < 0.01 vs Sham+Vehicle, *p* < 0.05 vs Sham+LM11A-31, Fig. 4c) compared to rats in the sham group. Rats that received LM11A-31 pretreatment showed lower levels of astrocyte activation in the cortex (*p* < 0.01, Fig. 4c) and hippocampus (*p* < 0.05, Fig. 4c) compared with the PM+Vehicle group. Furthermore, as shown in Fig. 4b, we found a significant increase in the expression of microglial markers Iba-1 in the cortex (*p* < 0.001 vs Sham+Vehicle, *p* < 0.001 vs Sham+LM11A-31, Fig. 4d) and hippocampus (*p* < 0.001 vs Sham+Vehicle, *p* < 0.001 vs Sham+LM11A-31, Fig. 4d) 24 h after infection in the PM+Vehicle group compared with the sham group. We also observed decreased expression of Iba-1 in the cortex (*p* < 0.001, Fig. 4d) and hippocampus (*p* < 0.01, Fig. 4d) in the PM+LM11A-31 group compared with the PM+Vehicle group.

### LM11A-31 pretreatment ameliorates neuronal apoptosis and necrosis of rats at 24 h after PM

**Fig. 5:**
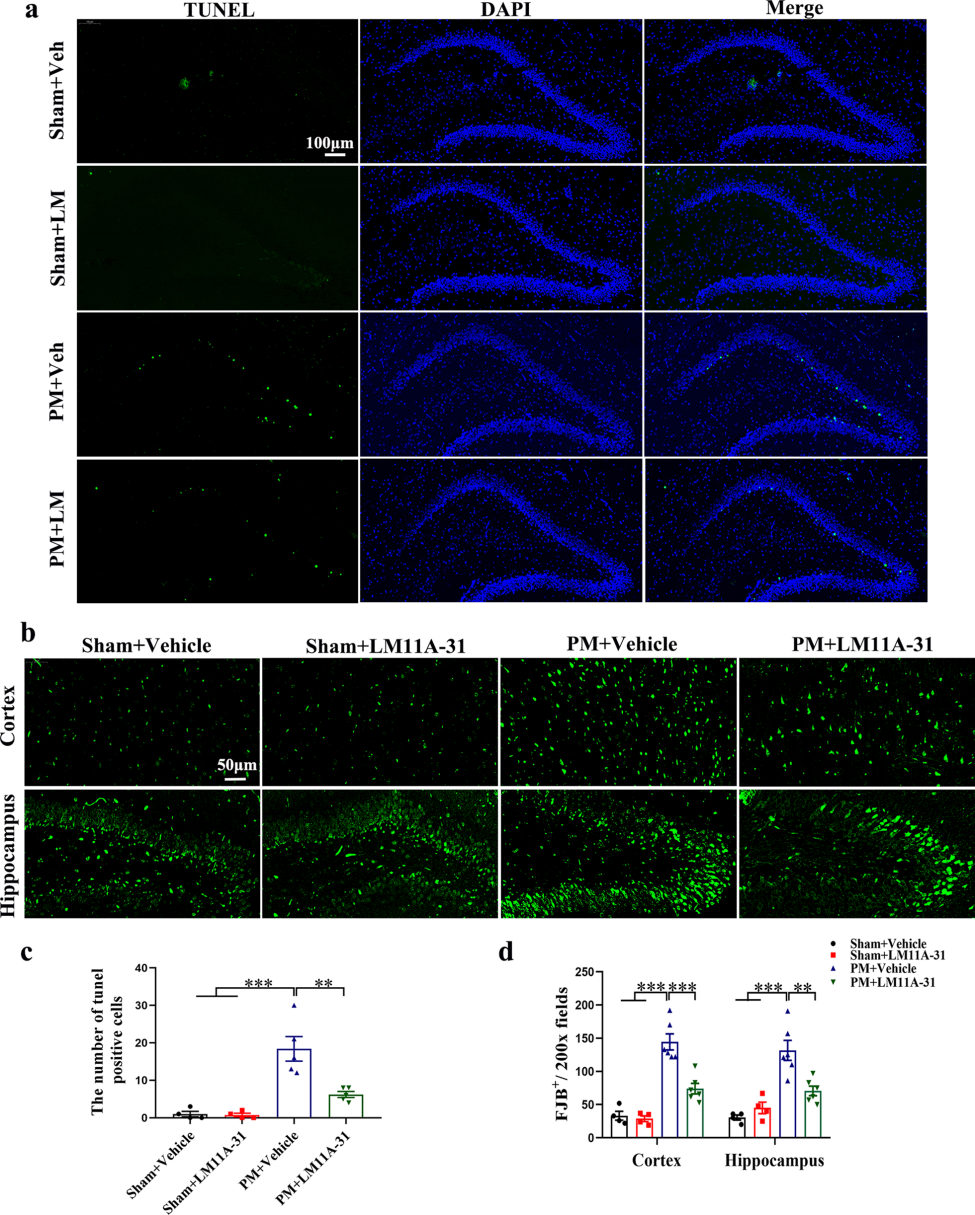
LM11A-31 pretreatment ameliorates neuronal apoptosis and necrosis at 24 h after PM induction. **a** Immunofluorescent staining showing apoptotic neurons assessed by TUNEL staining in the hippocampal DG. Scale bar = 100 μm. **b** Representative images showing degenerating neurons indicated by FluoroJdeB (FJB) staining in the cortex and hippocampal DG. Scale bar = 50 μm. **c** Quantification of TUNEL-positive cells in the hippocampal DG. **d** Quantification of FJB-positive degenerating cells/200× fields in the cortex and hippocampus. Data are presented as the mean ± SEM (*n* = 4–5 rats). **p < 0.01, ***p < 0.001

We detected neuronal apoptosis and necrosis using TUNEL and FJB staining, respectively. Compared with the sham group, significant hippocampal apoptosis was observed in the PM+Vehicle group (*p* < 0.001, Fig. 5a, c). Infected animals pretreated with LM11A-31 had significantly reduced hippocampal apoptosis than animals receiving vehicle pretreatment (*p* < 0.01, Fig. 5a, c). Moreover, there was significant neuronal necrosis in the cortex (*p* < 0.001, Fig. 5b, d) and hippocampus (*p* < 0.001, Fig. 5b, d) of rats in the PM+Vehicle group, compared with rats in the sham group. Intranasal pretreatment with LM11A-31 significantly decreased *S. pneumoniae*-induced increase in neuronal necrosis in the cortex (*p* < 0.001, Fig. 5b, d) and hippocampus (*p* < 0.01, Fig. 5b, d) compared with rats in the PM+Vehicle group.

### LM11A-31 pretreatment inhibits p75NTR and inflammation*-*related transcription factors (NF-κBp65, C/EBPβ) expression in the cortex and hippocampus of rats at 24 h after PM

**Fig. 6:**
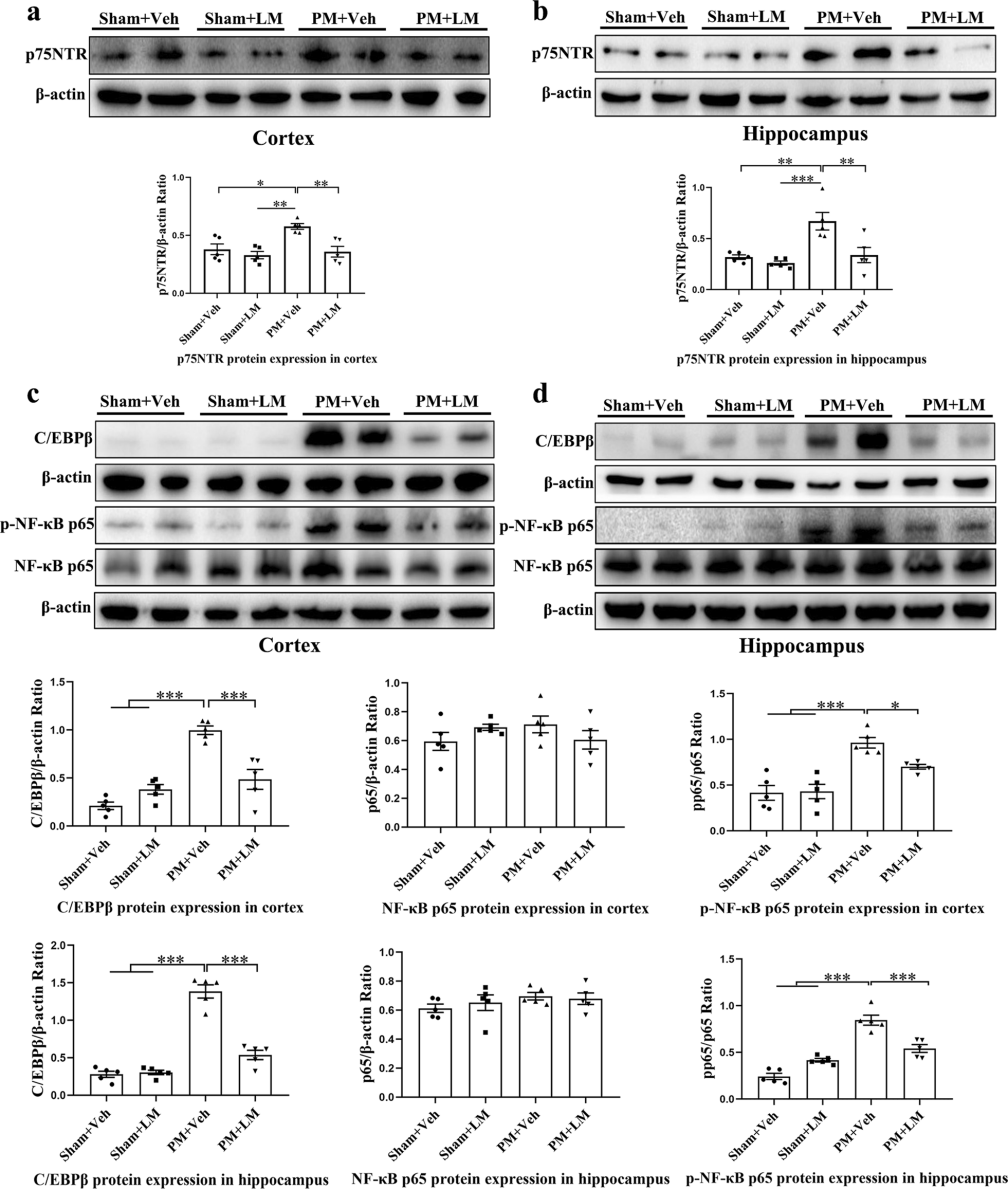
LM11A-31 pretreatment inhibits p75NTR and inflammation*-*related transcription factors expression at 24 h after PM induction. Western blot analysis of p75NTR, p-NF-κBp65/NF-κBp65 and C/EBPβ protein levels in the cortex **a**, **c** and hippocampus **b**, **d** from rats pretreated with vehicle or LM11A-31 at 24 h after PM induction. Proteins were normalized to *β*-actin. Data are presented as the mean ± SEM (*n* = 5 rats). *p < 0.05, **p < 0.01, ***p < 0.001

We evaluated p75NTR and two inflammation*-*related transcription factors (NF-κBp65, C/EBPβ) protein expression to examine whether LM11A-31-pretreatment could mediate the inhibitory effect of neuroinflammation by inhibiting the expression of p75NTR. As shown in Fig. 6a, b, LM11A-31 pretreatment caused a significant decrease of p75NTR protein expression in both the cortex (*p* < 0.01) and hippocampus (*p* < 0.01) of all infected rats. Furthermore, *S. pneumoniae* infection significantly increased the levels of p-NF-κBp65 and C/EBPβ in the cortex and hippocampus. Interestingly, administration of LM11A-31 partly inhibited p-NF-κBp65 and C/EBPβ expression in the cortex (^C/EBPβ^*p* < 0.001, ^p-NF-κB^*p* < 0.05, Fig. 6c) and hippocampus (^C/EBPβ^*p* < 0.001, ^p-NF-κB^*p* < 0.001, Fig. 6d) compared with levels observed in the PM+Vehicle group.

### LM11A-31 pretreatment downregulates the upregulation of proinflammatory cytokines/mediators (IL-1β, TNF-α, IL-6 and iNOS) levels in brain tissues and serum of rats at 24 h after PM

**Fig. 7:**
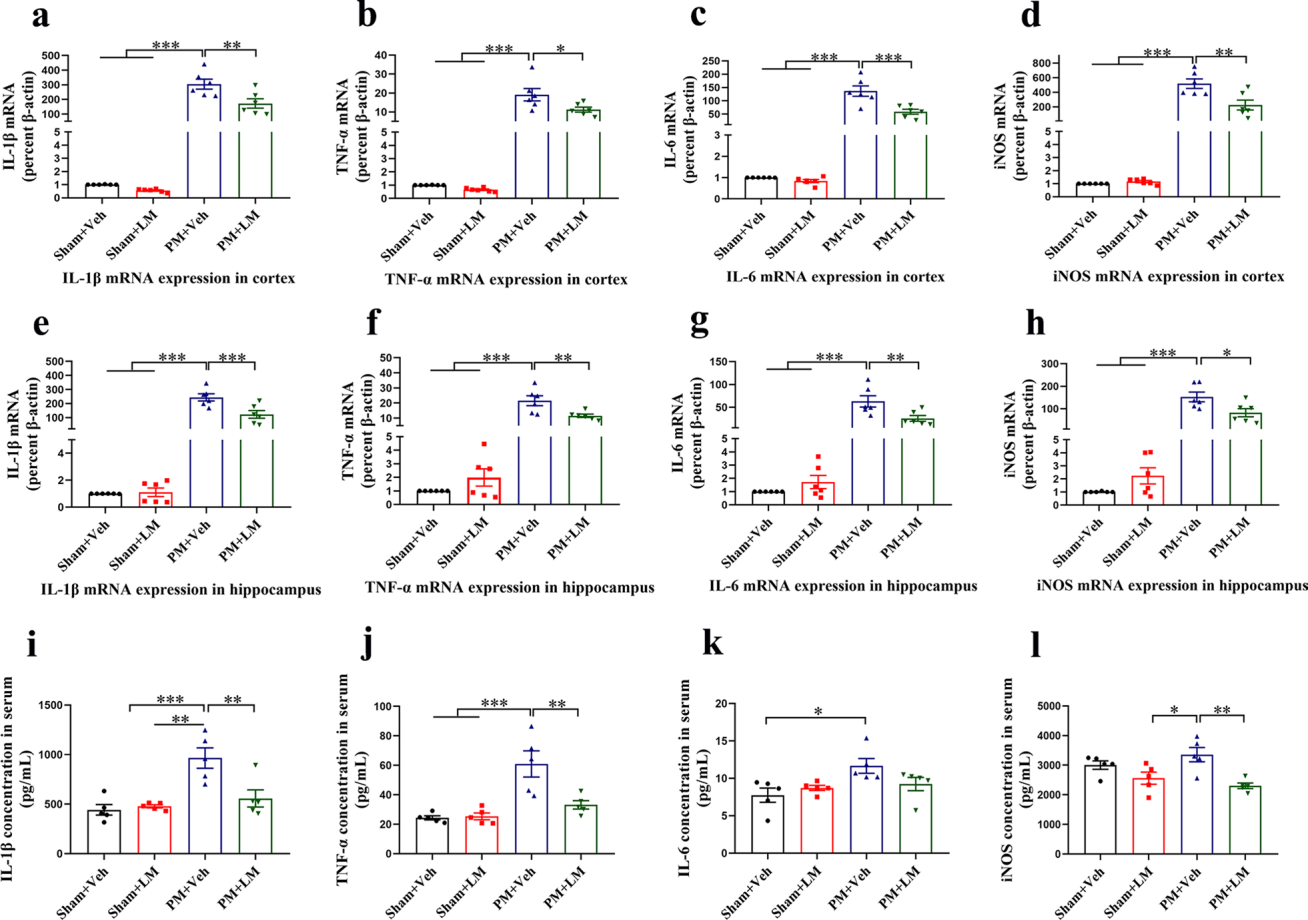
LM11A-31pretreatment downregulates proinflammatory cytokines/mediators expression at 24 h after PM induction. Levels of IL-1β **a**, **e**, **i**, TNF-α **b**, **f**, **j**, IL-6 **c**, **g, k**, and iNOS **d**, **h**, **l**, in brain tissues (cortex and hippocampus) and serum at 24 h post-infection were examined by RT-PCR and ELISA, respectively. Quantification of mRNA level expression was normalized to *β*-actin. Proinflammatory cytokines/mediators concentration in serum were expressed as pg/mL. Data are presented as the mean ± SEM (*n* = 5–6 rats). *p < 0.05, **p < 0.01, ***p < 0.001

Next, we measured the expression of proinflammatory cytokines/mediators (IL-1β, TNF-α, IL-6 and iNOS) in brain lysates (cortex and hippocampus) and serum at 24 h post-infection by RT-PCR and ELISA, respectively. As shown in Fig. 7a–l, the levels of all proinflammatory cytokines/mediators in brain lysates (cortex and hippocampus) and serum in the PM+Vehicle group were significantly higher than rats in the sham group. In addition, compared with the PM+Vehicle group, the levels of proinflammatory cytokines/mediators in the cortex (Fig. 7a–d) and hippocampus (Fig. 7e–h) and serum (Fig. 7i, j, l) in the PM+LM11A-31 group were significantly reduced. However, the expression level of IL-6 in serum showed no significant difference between the PM+Vehicle group and the PM+LM11A-31 group. Our results indicated that regulated p75NTR via intranasal administration of LM11A-31 could greatly ease the inflammatory storm triggered by PM.

### Knockdown of p75NTR in the cortex and hippocampus attenuates PM-induced brain injure

**Fig. 8:**
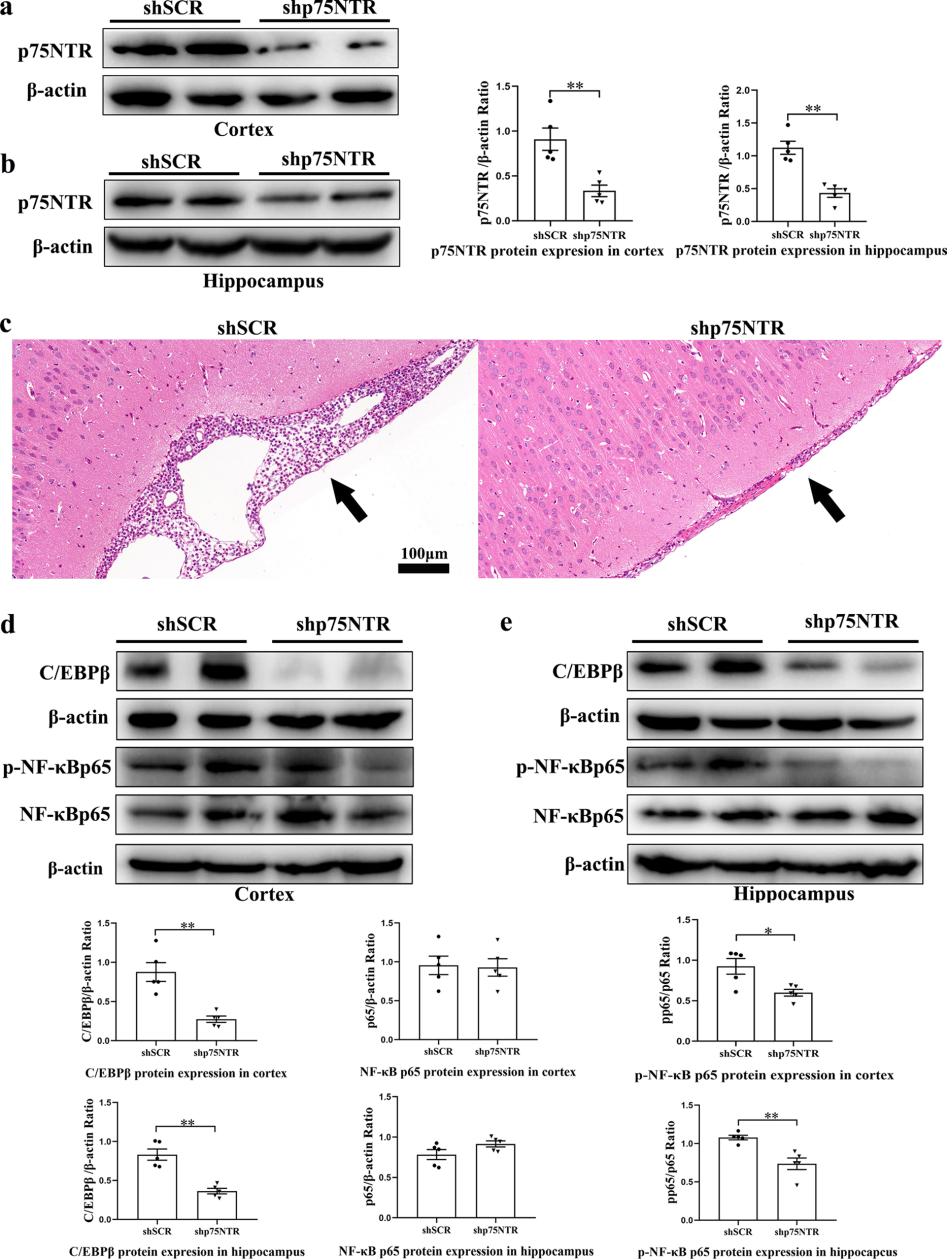
p75NTR knockdown reduces PM-induced brain injury. Ad-shp75NTR-mediated knockdown of p75NTR. One week after virus infusion, all rats were inoculated with *S. pneumoniae* in the lateral ventricle. **a**, **b** p75NTR protein levels in the cortex and hippocampus were measured by Western blot analysis 24 h post-infection. **c** Representative images of the sections with inflammatory cells in the cavum subarachnoidale. Scale bar = 100 μm. **d**, **e** Western blot analysis of inflammation*-*related transcription factors in the cortex and hippocampus from rats treated with shp75NTR or shSCR at 24 h after PM induction. Data are presented as the mean ± SEM (*n* = 5 rats). *p < 0.05, **p < 0.01

To further ascertain whether p75NTR participates in the neuroinflammation induction of PM, we developed an adenovirus expressing shRNA sequence targeting p75NTR (Ad-shp75NTR) and an adenovirus expressing a scrambled p75NTR shRNA sequence (Ad-shSCR). Next, we injected Ad-shp75NTR and Ad-shSCR into the right lateral ventricles of rats at 7 days before bacterial inoculation. At 24 h post-infection, the protein levels of p75NTR were significantly reduced in the cortex (*p* < 0.01, Fig. 8a) and hippocampus (*p* < 0.01, Fig. 8b) of rats infused with Ad-shp75NTR than the rats infused with Ad-shSCR. Meanwhile, coronal sections of the brain from two groups were evaluated by H&E staining. As shown in Fig. 8c, knockdown of p75NTR markedly alleviated subarachnoid expansion and reduced inflammatory cells infiltration. Additionally, we measured the levels of two inflammation*-*related transcription factors (NF-κBp65 and C/EBPβ) expression. Western blot analysis showed that knockdown of p75NTR significantly reduced the levels of p-NF-κBp65 and CEBP/β expression in the cortex (Fig. 8d) and hippocampus (Fig. 8e), consistent with the effect of LM11A-31 intervention on p75NTR. Thus, p75NTR in the brain contributes to the pathogenesis of PM.

### proBDNF/p75NTR pathway is activated in the hippocampus on days 7, 14, and 28 after PM

**Fig. 9:**
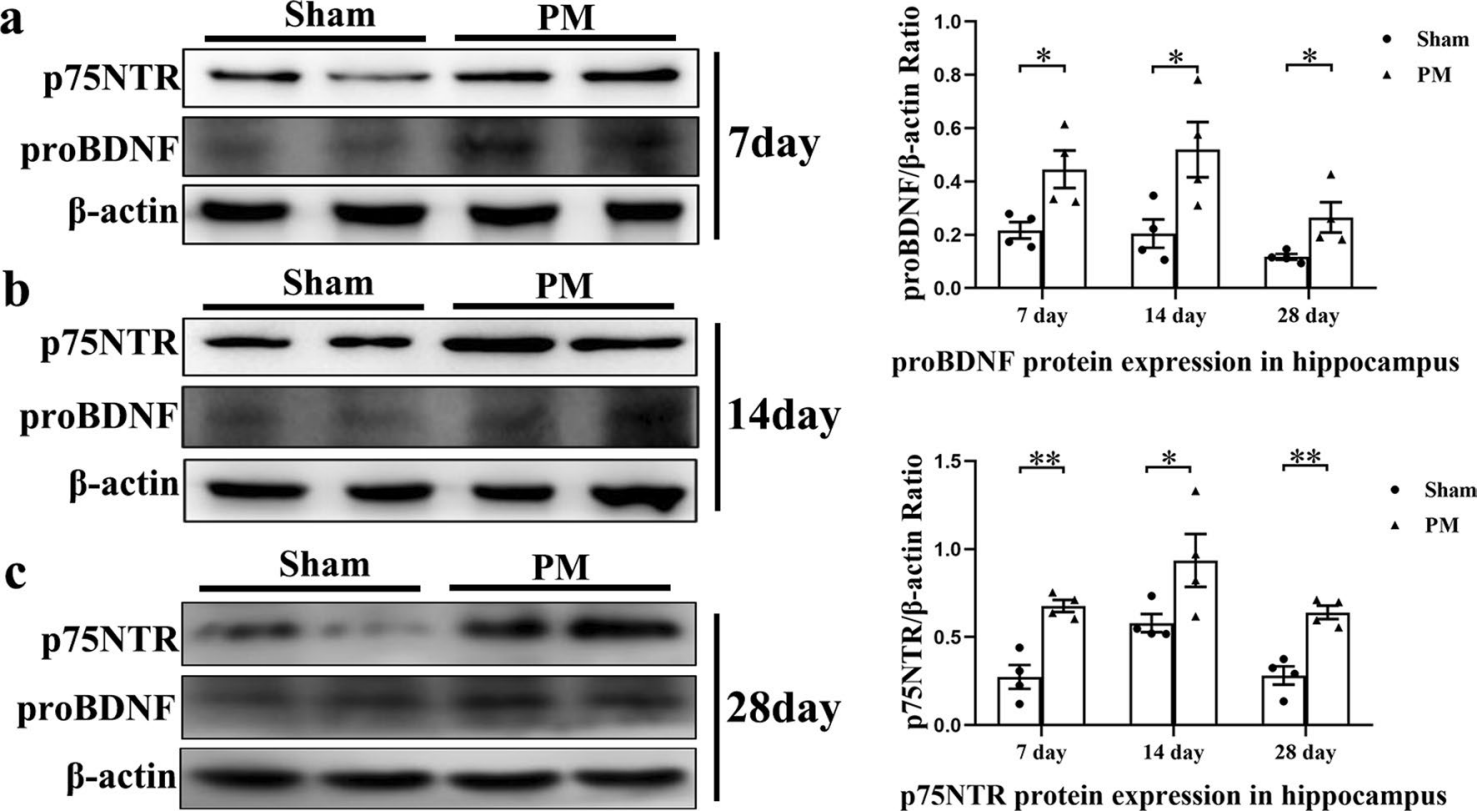
Changes in the expression of proBDNF/p75NTR after PM on days 7, 14 and 28. **a**–**c** The protein expressions of proBDNF and p75NTR in the hippocampus were measured on days 7, 14 and 28 after PM induction using Western blot. Proteins were normalized to *β*-actin. Data are presented as the mean ± SEM (*n* = 4 rats). *p < 0.05, **p < 0.01

After successfully inducing meningitis 24 h post-inoculation, ceftriaxone therapy was continued for the first 5 days. proBDNF and p75NTR proteins were analyzed using Western blot in the hippocampus on days 7, 14, and 28. We found that proBDNF and p75NTR protein levels significantly increased in hippocampus after 7 (^proBDNF^*p* < 0.05, ^p75NTR^*p* < 0.01, Fig. 9a), 14 (^proBDNF^*p* < 0.05, ^p75NTR^*p* < 0.05 Fig. 9b), and 28 (^proBDNF^*p* < 0.05, ^p75NTR^*p* < 0.01, Fig. 9c) days of meningitis induction, compared with rats in the sham group. These results suggest that the proBDNF/p75NTR signaling is persistently activated in the pathological process of PM.

### Dynamic changes in inflammation after long-term LM11A-31 treatment on days 7, 14 and 28

**Fig.10:**
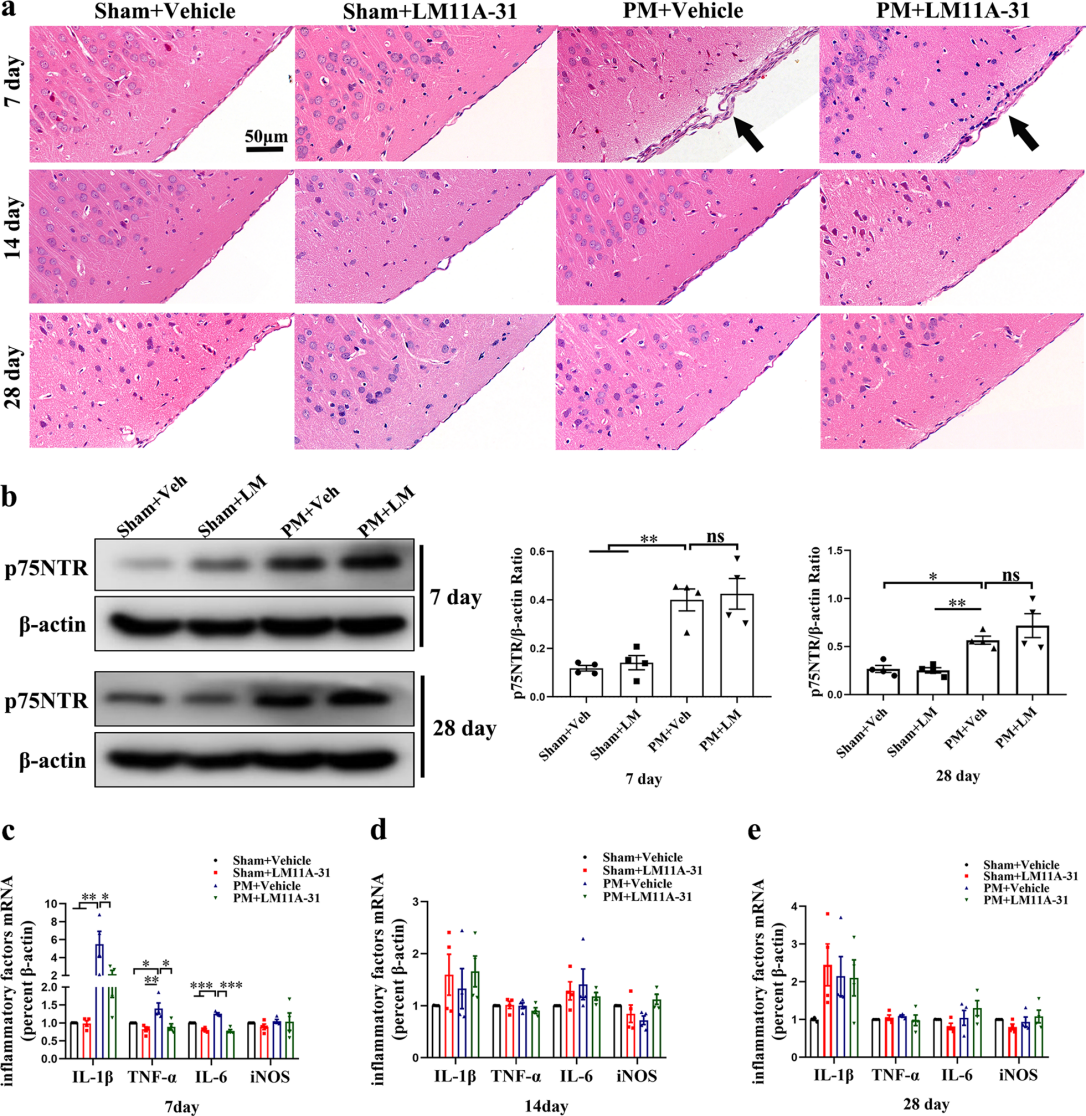
Dynamic changes in inflammation after long-term LM11A-31 treatment on days 7, 14 and 28. **a** Representative H&E staining of coronal brain sections from each group on days 7, 14 and 28. Scale bar = 50 μm. **b** Western blot analysis of p75NTR protein level in the hippocampus on days 7 and 28. Proteins were normalized to *β*-actin. **c**–**e** Relative mRNA expression of proinflammatory cytokines/mediators (IL-1β, TNF-α, IL-6 and iNOS) in the hippocampus on days 7, 14 and 28. Quantification of mRNA level expression was normalized to *β*-actin. Data are presented as the mean ± SEM (*n* = 4 rats). *ns* non-significant**,** *p < 0.05, **p < 0.01, ***p < 0.001

Since the proBDNF/p75NTR signaling is continuously activated on days 7, 14, and 28 after PM, we speculate whether long-term LM11A-31 intervention accelerates the resolution of inflammation after PM. Beyond 24 h post-infection, the survival rate of each group was recorded every day for 28 days. In the PM+Vehicle group, 7 out of 22 rats died within 6 days after 24 h of infection (3 on day 2, 3 on day 3 and 1 died on day 6). In the PM+LM11A-31 group, 5 out of 20 rats died within 7 days after 24 h of infection (3 on day 2, 1 on day 3 and 1 died on day 7). No deaths were observed after 7 days in all groups. However, there was no significant difference in survival rate between vehicle and LM11A-31-treated rats 24 h after infection (Additional file 2: Fig. S1, *p* = 0.6443). As shown in Fig. 10a, on day 7, infiltration of inflammatory cells was still observed in the subarachnoid space in all infected groups. Additionally, infected rats that received long-term LM11A-31 treatment showed a significant decrease in the number of inflammatory cells infiltrated compared with rats in the PM+Vehicle group. As time went by, inflammation effusion was eliminated in all infected groups on days 14 and 28. However, our Western blot results showed that the long-term treatment of LM11A-31 did not change the protein expression level of p75NTR in the hippocampus of PM rats (Fig. 10b). Then, we used RT-PCR  to assess the effects of LM11A-31 on the expression of proinflammatory cytokines/mediators (IL-1β, TNF-α, IL-6 and iNOS) induced by PM in the hippocampus. On day 7, the mRNA levels of IL-1β, TNF-α and IL-6 in the hippocampus were significantly elevated in the PM+Vehicle group than the rats in all the sham groups (Fig. 10c). As envisioned, the expression of inflammatory factors in the hippocampus was significantly reduced in the PM+LM11A-31 group than rats in the PM+Vehicle group. Similarly, there was no significant difference in the expression of proinflammatory cytokines/mediators in the hippocampus of each group at 14 and 28 days (Fig. 10d, e). Taken together, these results indicated that long-term LM11A-31 treatment accelerated the resolution of PM-induced inflammation.

### Long-term LM11A-31 treatment stimulates the proliferation of neuronal precursor cells of rats on days 7 and 14 after PM

**Fig. 11:**
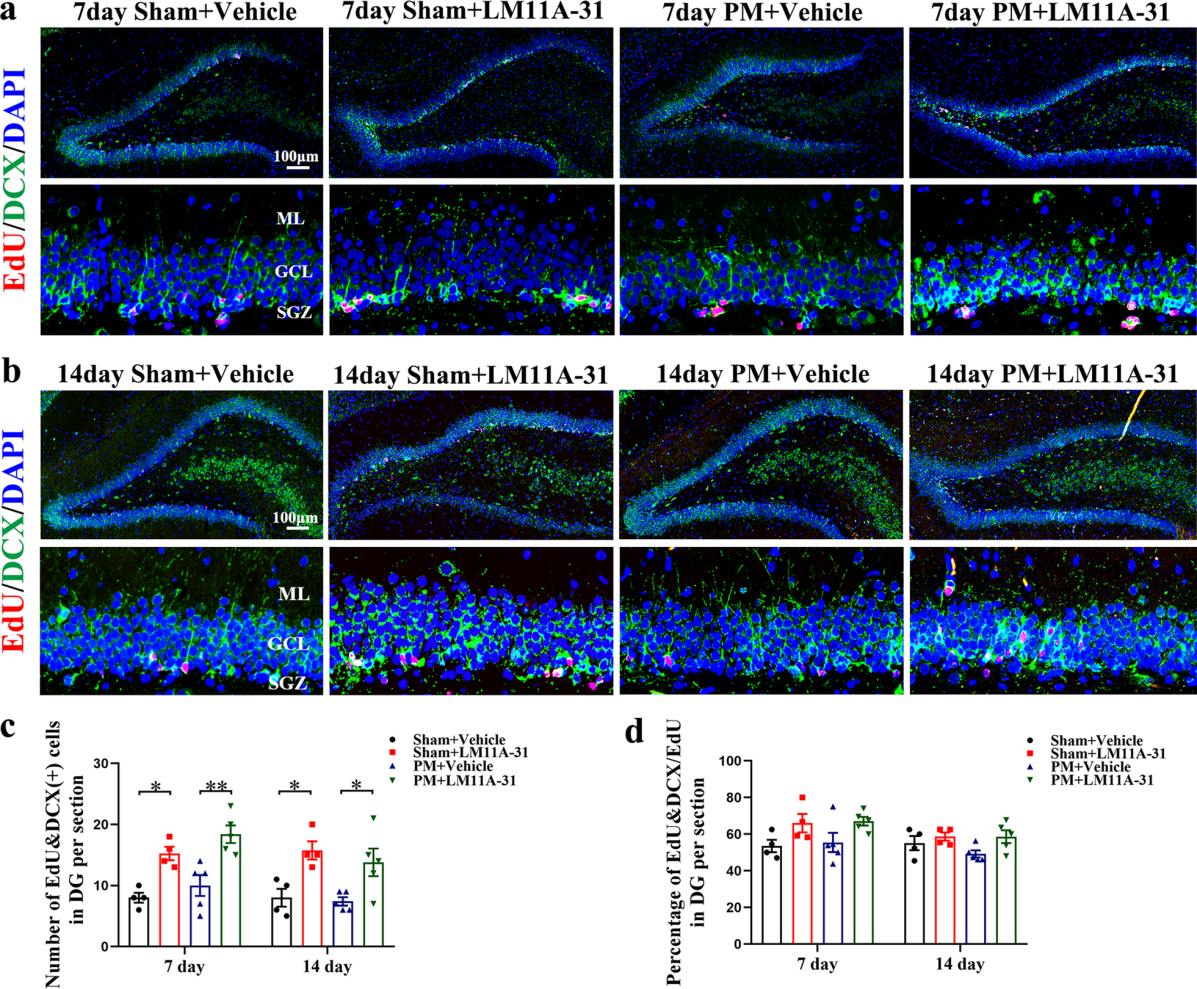
Long-term LM11A-31 treatment promotes neuronal precursor cells proliferation on days 7 and 14. **a**, **b** Representative images of DCX (green)/EdU (red) cells in the hippocampal DG at 7 and 14 days after PM. Scale bar = 100 μm. **c** Quantification of newly formed neuronal precursor cells (EdU^+^/DCX^+^) in the hippocampal DG at 7 and 14 days. **d** Quantification of newly formed neuronal precursor cells (EdU^+^/DCX^+^), as a percent of total EdU^+^ in the hippocampal DG at 7 and 14 days. Data are presented as the mean ± SEM (*n* = 4–5 rats). *p < 0.05, **p < 0.01

After 7 consecutive days of adjuvant treatment with LM11A-31 or vehicle, the expression of new neuronal progenitor cells, namely EdU&DCX double-positive cells in hippocampal DG region, were detected by immunofluorescence double-labeling method on days 7 (Fig. 11a) and 14 (Fig. 11b). We also analyzed the ratios of EdU&DCX double-positive cells relative to the total EdU positive cells. The quantification of data shown that the expression levels of EdU&DCX double-positive cells in the sham rats were upregulated by LM11A-31 treatment at 7 (*p* < 0.05, Fig. 11c) and 14 (*p* < 0.05, Fig. 11c) days. Interestingly, compared with the Sham+Vehicle group, the number of EdU&DCX double-positive cells increased slightly in the vehicle-treated rats at 7 days following PM injury. This may be due to the restrictive number of rats in each group, this rise, however, did not reach a significant level as in our previous study [40]. In addition, following PM injury, LM11A-31 treatment was associated with a significant increase of the EdU&DCX double-positive cells compared with vehicle-treated rats at 7 (*p* < 0.01, Fig. 11c) and 14 (*p* < 0.05, Fig. 11c) days. However, the treatment of LM11A-31 did not significantly change the percentage of EdU&DCX/EdU cells in either sham groups or PM groups at 7 and 14 days (Fig. 11d).

### Long-term LM11A-31 treatment promotes the formation of mature neurons of rats on day 28 after PM

**Fig. 12:**
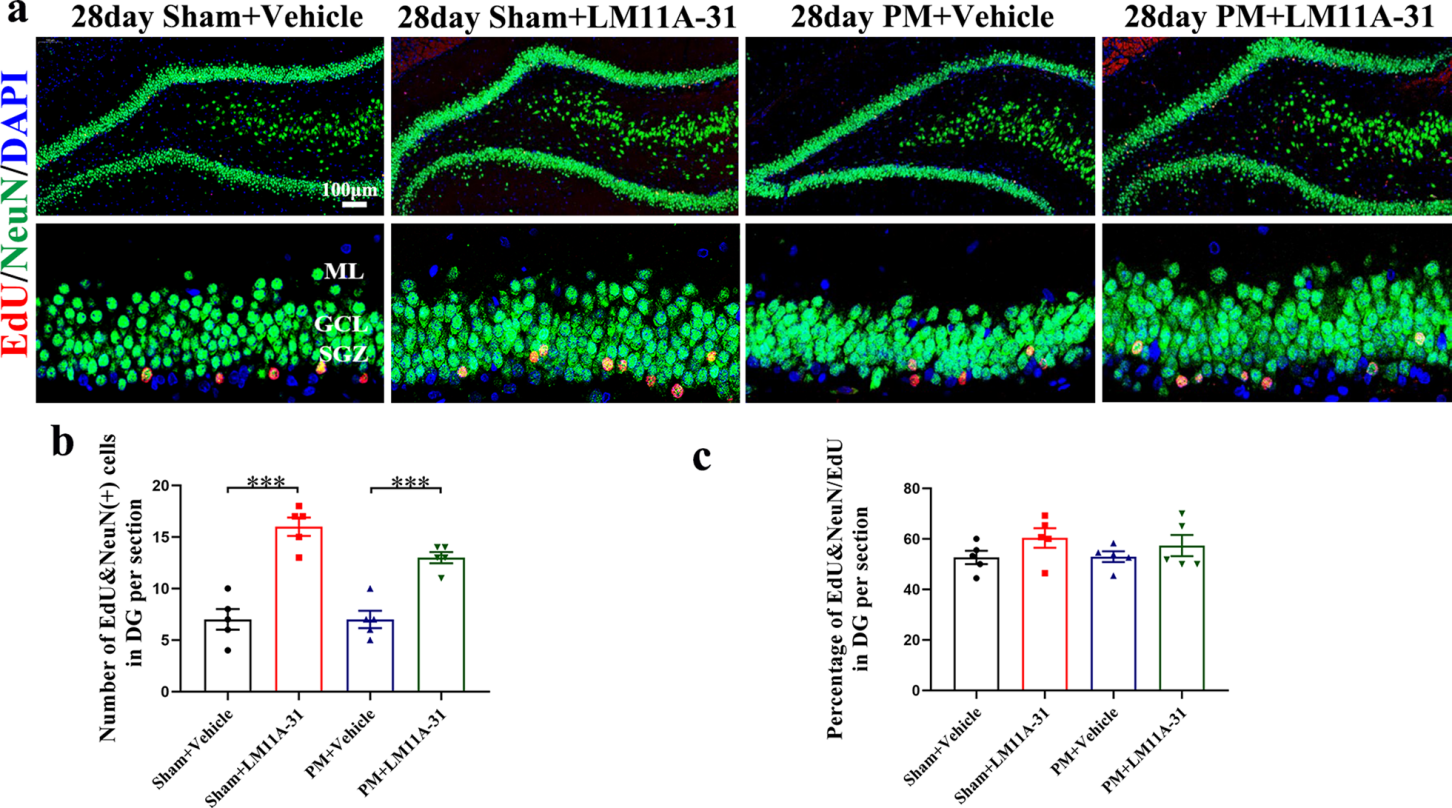
Long-term LM11A-31 treatment promotes neurogenesis on day 28. **a** Representative image of NeuN (green)/EdU (red) cells in the hippocampal DG 28 days after PM. Scale bar = 100 μm. **b** Quantification of newly formed mature neuron cells (EDU^+^/NeuN^+^) in the hippocampal DG at 28 days. **c** Quantification of newly formed neuronal precursor cells (EdU^+^/DCX^+^), as a percent of total EdU^+^ in the hippocampal DG at 28 days. Data are presented as the mean ± SEM (*n* = 5 rats). ***p < 0.001

To determine whether early LM11A-31 effects on precursor populations would lead to long-term changes, the numbers of EdU&NeuN double-positive cells were examined on day 28 after infection. We found that most of the EdU^+^ cells were expressed in SGZ and GCL of the DG (Fig. 12a). Treatment with LM11A-31 produced a significant increase in the number of EdU&NeuN double-positive cells in the sham (*p* < 0.001, Fig. 12b) and PM (*p* < 0.001, Fig. 12b) groups relative to the vehicle-treated group. We further found that the percentage of EdU&NeuN/EdU cells was not significantly different between the sham and PM groups and rats which were treated with LM11A-31 or vehicle (Fig. 12c).

## Discussion

In the present study, we observed that p75NTR, as a negative modulator, plays an important role in the pathophysiology of PM. In acute PM, p75NTR is upregulated in the cortex and hippocampus, LM11A-31 pretreatment reduced the clinical and pathological severity, inhibited neuroinflammatory cascade, and ameliorated neuronal damage and apoptosis in rats with PM. We further identified that blocking p75NTR signaling in PM with shRNA-mediated knockdown of the receptor, also blunted *S. pneumoniae*-induced neuroinflammation. In addition, our study also found that proBDNF/p75NTR signaling was persistently activated on days 7, 14, and 28, after infection, and long-term treatment of LM11A-31 accelerated the resolution of PM-induced inflammation, and had a positive effect on the proliferation of neuronal precursor cells and the formation of mature neurons of PM. To our knowledge, this report is the first to demonstrate that modulation of p75NTR attenuates neuroinflammation and promotes hippocampal neurogenesis in experimental PM.

Despite implementing effective treatments and modern intensive care facilities for PM, the rate of mortality and morbidity as well as long-lasting neurological sequelae rates remain high [3, 41]. Brain damage caused by PM is mainly correlated with an exacerbated inflammation response, which is not simply due to the rapid growth of *S. pneumoniae* in the subarachnoid space but is also largely dependent on the overshooting host immune response [4]. Additionally, bacteriolytic antibiotics therapy results in rapid bacterial cell lysis and a sudden release of large amounts of immunogenic bacterial components may even transiently exacerbate the host immune response [42, 43]. A recent study reported that ceftriaxone monotherapy caused a transient increase of proinflammatory cytokines (IL-1β, IL-6) in the CSF in experimental PM [44]. This phenomenon may occur due to the rapid release of antibiotic-induced highly immunogenic bacterial lysis products and therefore aggravated the inflammation. Current anti-infective therapy to prevent PM-induced injury has limited efficacy. Understanding the mechanisms behind *S. pneumoniae*-induced injury is key for developing new and effective adjuvant therapeutic targets for PM. In recent years, increasing evidence found that proBDNF/p75NTR signaling has emerged as a major contributor to many pathophysiological processes in the CNS [23, 45, 46]. The opposing nature of BDNF/TrkB and proBDNF/p75NTR prompted us to hypothesize that BDNF/TrkB and proBDNF/p75NTR might serve as protection and damage signals in PM, respectively.

During acute PM, we examined proBDNF/p75NTR expression in the cortex and hippocampus upon *S. pneumoniae*-induced neuroinflammation. Although the protein level of proBDNF did not change significantly, the p75NTR protein level was increased. Notably, the increased expression of p75NTR was mainly found in neurons and astrocytes, which is in line with previously reported studies [47, 48]. In addition to neurons and astrocytes, p75NTR was also detected on various immune cells, including peripheral mononuclear blood cells and B lymphocytes, mediating a series of neuroinflammatory responses [35, 49]. Furthermore, Choi et al. reported that IL-1β and TNF-α upregulate p75NTR expression in neurons and astrocytes via the p38MAPK and NF-κB pathway, causing glial cell proliferation and neuronal cell death [33]. Importantly, these cytokines have also been identified to be vital mediators in the course of *S. pneumoniae* infection [50]. Although the role of p75NTR in inflammation has not been fully elucidated, the above findings suggest that p75NTR may have a potential effect on the inflammation and neuronal damage of PM.

Next, we pretreated rats with non-invasive nasal administration of LM11A-31 or vehicle. The dose of LM11A-31 (15 μg/day) was chosen based on a preliminary pilot study and previous studies [39, 51]. In the process of *S. pneumoniae* infection, the activated microglia adapt to amoeba-like phenotype, initiate an inflammatory response, and then release proinflammatory factors such as IL-1 β and TNF-α, which further activate astrocytes and magnify the inflammatory neural network, resulting in an increased inflammatory response and neuronal injury [52, 53]. In the present study, pretreatment of LM11A-31 was linked with inhibition of both astrocytic and microglial activation in the cortex and hippocampus. Since there was no expression of p75NTR in microglia in the PM model, the anti-inflammation effects of LM11A-31 might have been indirect, principally via its ability to attenuate the amplification of the inflammatory network and injured neurons. Interestingly, Meeker et al. reported that the response of microglia to LM11A-31 may be secondary to neurons changes in the feline immunodeficiency virus-mediated neuronal injury model, rather than microglia themselves, which supports our speculation [54]. In addition, activated astrocytes and microglia in the CNS are related to the transcription of inflammation-related genes and the production of proinflammatory cytokines. The results from this study demonstrate the activation of inflammation-related transcription factors (NFκB, C/EBPβ) and a storm of inflammatory cytokines in the cortex and hippocampus of PM rats, consistent with previous findings [55, 56]. NF-kB p65 is a critical transcription factor of many proinflammatory genes involved in the pathogenesis of experimental PM. Activation of NF-kB p65 is a hallmark of the inflammatory host response [55]. Koedel et al. reported that the pharmacologic interference with NF-kB p65 activation reduced meningitis-related inflammatory response and intracranial complications in experimental pneumococcal meningitis [56]. Moreover, the research of the C/EBP family in neuroinflammation has become a hot spot in recent years. Increasing evidence has shown that C/EBPβ plays a vital role in neuroinflammation [57, 58]. Straccia et al. found that the absence of C/EBPβ in glial cultures inhibits inflammatory-related gene expression and attenuates neuronal loss induced by activated microglia [59]. C/EBPβ null mice showed a reduced inflammatory response following kainic acid-triggered brain injury and transient focal cerebral ischemia injury [60, 61]. These results reveal the essential function for NFκB and C/EBPβ in CNS inflammatory injury. Our study found that pretreatment with LM11A-31 to inhibit the expression of p75NTR significantly reduced the activation of two inflammation-related transcription factors of PM rats, and then reduced the expression of inflammatory factors in the cortex and hippocampus. PM-induced neuronal necrosis and apoptosis were also significantly reduced by LM11A-31 pretreatment. These results suggest that the regulation of p75NTR by pretreatment with LM11A-31 can protect neurons from PM-related inflammation injury. In addition, adenovirus-mediated knockdown of p75NTR also alleviated neuroinflammatory injury in PM rats, suggesting an important role for p75NTR in the pathogenic mechanism of PM. Noteworthy, the reports on p75NTR signaling as inflammatory mediators are very limited, and the co-receptors as well as downstream pathways related to the inflammation action of p75NTR are challenging to determine due to their diversity and complex interactions. Therefore, further studies should be designed to explore the potential mechanisms underlying the pro-inflammatory effect of p75NTR in PM.

Compared with the auxiliary anti-inflammatory agents given during acute PM, promoting endogenous hippocampal neurogenesis to replace the lost neurons is another effective strategy to improve the prognosis after pediatric PM. Among them, the role of a neurotrophic signaling pathway in neuronal regeneration has gained more attention. Many studies suggested that the increased expression of BDNF and TrkB would enhance neurogenesis and neuronal survival [22, 62]. Contradictorily, proBDNF binds to p75NTR, causing an increase in neuronal apoptosis, neuronal migration reduction, and neuronal regeneration inhibition [23]. The results of these subtle neurotrophic dysregulations may be the basis of learning and memory dysfunction in pediatrics PM. Our previous study found that the expression of BDNF and TrkB decreased significantly after antibiotic treatment for PM [63, 64]. Furthermore, exogenous BDNF could promote the proliferation of endogenous NSC, while the newly generated cells were not well integrated into the hippocampal circuitry to form mature neurons [22]. Interestingly, in the present study, we found that the expression of proBDNF and p75NTR continued to upregulate in the post-acute phase of PM. These results indicated that the balance between BDNF/TrkB pathway and the proBDNF/p75NTR pathway was broken. The failure of exogenous BDNF to improve the formation of mature neurons in PM may be due to the increase of proBDNF and its high affinity receptor p75NTR. Certainly, we cannot rule out the possible effects of other signal transduction pathways related to endogenous neurogenesis, for example Notch, Wnt [65, 66]. Previous studies have shown that persistent inflammatory cascades change the neurogenic microenvironment, driving activation of cell death machinery in immature neurons [67, 68]. Regulation of p75NTR by treatment with LM11A-31 can reduce neuroinflammatory response and inhibit p75NTR-associated apoptotic signaling [47, 69]. Furthermore, p75NTR has been considered as a direct regulator of neurogenesis [70]. LM11A-31 may directly activate neurotrophic signaling in cells expressing p75NTR [71]. Thus, we speculated that regulating p75NTR signaling by LM11A-31 may improve endogenous neurogenesis of pediatric PM. In the present study, after delivering intranasally LM11A-31 or vehicle for 7 consecutive days, we detected the dynamic changes in inflammation, the number and percentage of EdU&DCX cells on days 7 and 14, as well as EdU&NeuN cells on day 28 after infection in the hippocampus of all groups. We found that long-term LM11A-31 treatment accelerated the resolution of PM-induced inflammation. In addition, early differentiation of NSCs, known as the number of EdU&DCX, was increased on days 7 and 14 after long-term LM11A-31 treatment, and its effect lasts until 28 days after injury, resulting in a significant increase in numbers of EdU/NeuN cells, compared to vehicle-treated rats. However, whether under sham or PM conditions, the percentage of DCX or NeuN expression in EdU cells increased slightly after long-term LM11A-31 treatment but did not significantly different compared to the vehicle treatment. Taken together, LM11A-31 causes the recovery of neurogenesis mainly by increasing the proliferation and/or survival rate of neuronal precursors, consistent with the results of Shi et al. in the TBI model [39]. Furthermore, whether LM11A-31 can induce neuron proliferation by regulating the differentiation of NSCs still needs to be elucidated. These results suggest that p75NTR is a potential regulatory target of PM neurogenesis. Given the limitations of BDNF in promoting neurogenesis found in our previous study [22], modulation of p75NTR signaling may potentially improve the chance of successful neuroregeneration after hippocampal injury induced by PM.

Certain limitations of the present study need to be considered. First, given the limited volume of CSF that could be harvested from infected rats, the anti- inflammatory effect of LM11A-31 couldn’t be determined in the CSF. Second, although we found that the proBDNF/p75NTR signaling may serve as a negative regulatory after PM, we cannot ignore the potential interaction between other proneurotrophins (i.e., proNGF and proNT3) and p75NTR in the pathology of PM. In addition, while our data identify the important role of p75NTR in PM, future studies are needed to fully understand its specific molecular mechanism in regulating inflammation and neurogenesis.

## Conclusions

Anti-inflammation and promoting neurogenesis are assumed as two effective ways to improve the outcome of PM. To the best of our knowledge, we show for the first time that p75NTR was consistently activated during PM, and both anti-inflammatory and pro-neurogenic effects were confirmed after modulating p75NTR. Specifically, in the acute phase of PM, modulation of p75NTR signaling by pretreatment with LM11A-31 prevented PM-induced injury, including inhibited astrocytes and microglia activation, reduced expression of inflammation*-*related transcription factors (NF-κB, C/EBPβ) and proinflammatory cytokines/mediators (IL-1β, TNF-α, IL-6 and iNOS), and ameliorated neuronal apoptosis and necrosis. In addition, genetic inactivation further confirmed the detrimental role of p75NTR in PM-induced neuroinflammatory injury. In the long-term phase of PM, LM11A-31 treatment accelerated inflammation resolution and, more importantly, promoted hippocampal neurogenesis. Overall, our findings provide strong evidence that regulating p75NTR, might open novel avenues for therapeutic intervention in PM-induced injury.

## Supplementary Information

**Additional file 1: Table 1.** Sequences of PCR primers.

**Additional file 2: Figure S1.** Kaplan–Meier curves showing the survival rates of rats from different groups beyond 24 h post-infection.

## Data Availability

All data generated or analyzed during this study are included in this published article and its Additional files 1, 2.

## Abbreviations

proBDNF: Precursor brain-derived neurotrophic factor
p75NTR: p75 Neurotrophin receptor
TrkB: Tropomyosin-receptor kinase B
BM: Bacterial meningitis
PM: Pneumococcal meningitis
*S. pneumoniae*: *Streptococcus pneumonia*
CNS: Central nervous system
BBB: Blood–brain barrier
CSF: Cerebrospinal fluid
DMSO: Dimethyl sulfoxide
IL: Interleukin
TNF-α: Tumor necrosis factor-α
iNOS: Inducible nitric oxide synthase
NF- κB: Nuclear factor kappa B
RT-PCR: Real-time polymerase chain
TBST: Tris-buffered saline and Tween-20
TUNEL: Tissue pathology and terminal deoxynucleotidyl transferase dUTP-nick-end labeling
FJB: FluoroJade B
C/EBPβ: CCAAT/enhancer binding protein beta
EdU: Ethynyl deoxyuridine
DG: Dentate gyrus
SGZ: Subgranular zone
GCL: Granule cell layer
ML: Molecular layer
NSC: Neural stem cells
TBI: Traumatic brain injury
LTP: Long-term potentiation
HD: Huntington’s disease
IHC: Intracerebral hemorrhage
