# Supplementary material for: Regulation of the p75 neurotrophin receptor attenuates neuroinflammation and stimulates hippocampal neurogenesis in experimental Streptococcus pneumoniae meningitis
Source: J Neuroinflammation. 2021 Nov 2;18:253. doi: 10.1186/s12974-021-02294-w (PMC8561879; doi:10.1186/s12974-021-02294-w)
Supplement: Supplementary file 1 — Additional file 1: Table 1. Sequences of PCR primers. [file 12974_2021_2294_MOESM1_ESM.docx]

**Additional Table 1. Sequences of PCR primers**

| **Gene** | **Primer** |
| --- | --- |
| **β-actin** | Sense primer: 5′- CACCCGCGAGTACAACCTTC -3′  Antisense primer: 5′- CCCATACCCACCATCACACC -3′ |
| **TNF-α** | Sense primer: 5′-ATGGGCTCCCTCTCATCAGTTCC-3′  Antisense primer: 5′-GCTCCTCCGCTTGGTGGTTTG-3′ |
| **IL-6** | Sense primer: 5′-ACTTCCAGCCAGTTGCCTTCTTG-3′  Antisense primer: 5′-TGGTCTGTTGTGGGTGGTATCCTC-3′ |
| **IL-1β** | Sense primer: 5′-CTCACAGCAGCATCTCGACAAGAG-3′  Antisense primer: 5′-TCCACGGGCAAGACATAGGTAGC-3′ |
| **iNOS** | Sense primer: 5′-GAGACGCACAGGCAGAGGTTG-3′  Antisense primer: 5′-AGCAGGCACACGCAATGATGG-3′ |
|  |  |
