# Supplementary figures and images for: Regulation of the p75 neurotrophin receptor attenuates neuroinflammation and stimulates hippocampal neurogenesis in experimental Streptococcus pneumoniae meningitis
Source: J Neuroinflammation. 2021 Nov 2;18:253. doi: 10.1186/s12974-021-02294-w (PMC8561879; doi:10.1186/s12974-021-02294-w)

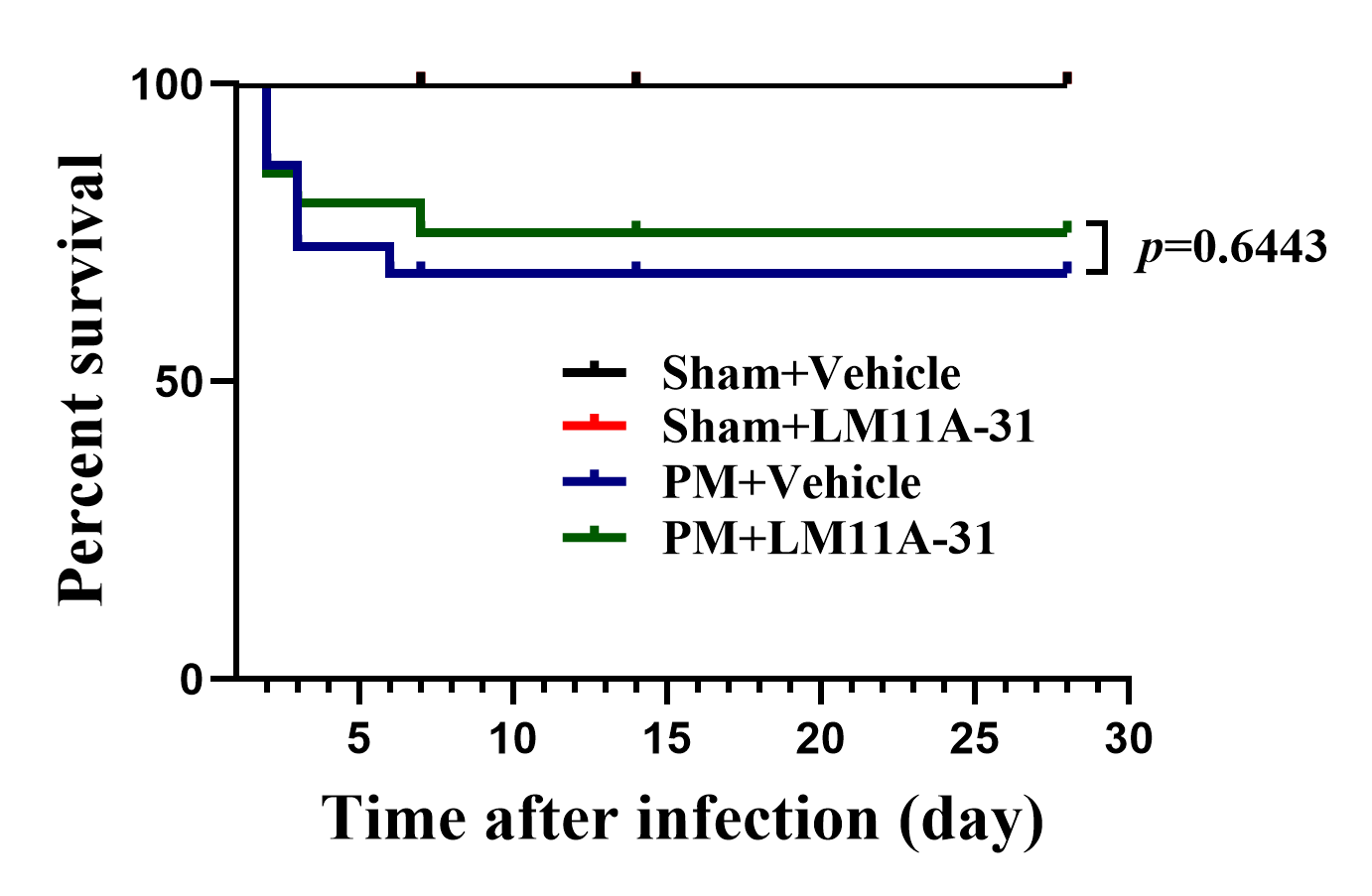

Supplement: Supplementary file 2 — Additional file 2: Figure S1. Kaplan–Meier curves showing the survival rates of rats from different groups beyond 24 h post-infection. [file 12974_2021_2294_MOESM2_ESM.tif]
